# Allelic Variation and Transcriptional Isoforms of Wheat *TaMYC1* Gene Regulating Anthocyanin Synthesis in Pericarp

**DOI:** 10.3389/fpls.2017.01645

**Published:** 2017-09-21

**Authors:** Yuan Zong, Xinyuan Xi, Shiming Li, Wenjie Chen, Bo Zhang, Dengcai Liu, Baolong Liu, Daowen Wang, Huaigang Zhang

**Affiliations:** ^1^State Key Laboratory of Plateau Ecology and Agriculture, Qinghai University Xining, China; ^2^Key Laboratory of Adaptation and Evolution of Plateau Biota, Northwest Institute of Plateau Biology, Chinese Academy of Sciences Xining, China; ^3^University of Chinese Academy of Sciences Beijing, China; ^4^Triticeae Research Institute, Sichuan Agricultural University Chengdu, China; ^5^State Key Laboratory of Plant Cell and Chromosome Engineering, Institute of Genetics and Developmental Biology, Chinese Academy of Sciences Beijing, China

**Keywords:** common wheat, purple pericarp, anthocyanin biosynthesis, bHLH transcription factor, *Pp3*

## Abstract

Recently the *TaMYC1* gene encoding bHLH transcription factor has been isolated from the bread wheat (*Triticum aestivum* L.) genome and shown to co-locate with the *Pp3* gene conferring purple pericarp color. As a functional evidence of *TaMYC1* and *Pp3* being the same, higher transcriptional activity of the *TaMYC1* gene in colored pericarp compared to uncolored one has been demonstrated. In the current study, we present additional strong evidences of *TaMYC1* to be a synonym of *Pp3*. Furthermore, we have found differences between dominant and recessive *Pp3*(*TaMyc1*) alleles. Light enhancement of *TaMYC1* transcription was paralleled with increased AP accumulation only in purple-grain wheat. Coexpression of *TaMYC1* and the maize MYB TF gene *ZmC1* induced AP accumulation in the coleoptile of white-grain wheat. Suppression of *TaMYC1* significantly reduced AP content in purple grains. Two distinct *TaMYC1* alleles (*TaMYC1p* and *TaMYC1w*) were isolated from purple- and white-grained wheat, respectively. A unique, compound *cis*-acting regulatory element had six copies in the promoter of *TaMYC1p*, but was present only once in *TaMYC1w*. Analysis of recombinant inbred lines showed that *TaMYC1p* was necessary but not sufficient for AP accumulation in the pericarp tissues. Examination of larger sets of germplasm lines indicated that the evolution of purple pericarp in tetraploid wheat was accompanied by the presence of *TaMYC1p*. Our findings may promote more systematic basic and applied studies of anthocyanins in common wheat and related Triticeae crops.

## Introduction

Anthocyanin pigments (APs) constitute an important class of secondary metabolites synthesized by most plants. They are responsible for the pigmentation of different types of plant organs, and function as attractors for the vectors of pollens and seeds (Joaquin-Cruz et al., 2015). APs have been found to participate in non-specific disease resistance and the protection against biotic and abiotic stresses in plants (Treutter, 2006). Consequently, adverse environmental factors, such as high light, low temperature, high salinity, and/or drought stress, generally induce AP accumulation (Jayalakshmi et al., 2012). In recent years, anthocyanins have attracted wide attention owing to their anti-inflammatory, anti-mutagenic, anti-carcinogenic, and anti-bacterial effects (Mazza, 2007; Wang and Stoner, 2008; Bowen-Forbes et al., 2010). In common wheat (*Triticum aestivum*, 2*n* = 6x = 42, AABBDD) and durum wheat (*T. turgidum* ssp. *durum*, 2*n* = 4x = 28, AABB) crops, APs accumulated in the grains represent a valuable source of dietary bioactive materials in the functional food industry (Li et al., 2007; Khlestkina et al., 2011; Revanappa and Salimath, 2011).

Anthocyanin biosynthesis and metabolic pathways have been studied in many plant species. The main structural genes for anthocyanin biosynthesis encode phenylalanine ammonia lyase, chalcone synthase, chalcone isomerase, flavanone 3-hydroxylase, flavonoid 3-hydroxylase, dihydroflavonol 4-reductase, leucoanthocyanidin dioxygenase, and flavonoid 3-O-glucosyltransferase (Holton and Cornish, 1995). Two major classes of transcription factors (TFs), MYB and basic helix-loop-helix (bHLH), have been found to regulate the expression of anthocyanin biosynthesis genes (Zhang et al., 2014). Allelic variations of a number of MYB and bHLH TFs have been linked with differential accumulations of APs in different plant organs (Zhang et al., 2014). The MYB TF ZmC1 is well-known for its role in regulating anthocyanin biosynthesis in maize (McClintock, 1950; Pazares et al., 1987). DNA sequence variation in the promoter region of a functional MYB TF gene, *VvmybA1*, leads to changes in the flesh pigment content of grapes (Kobayashi et al., 2005; Porret et al., 2006; This et al., 2007). Similar MYB regulators have also been characterized in *Arabidopsis* (*PAP1* and *PAP2*) (Borevitz et al., 2000), petunia (*MYB3*) (Solano et al., 1995), and sweet potato (*MYB10*) (Feng et al., 2010). The bHLH TFs affecting anthocyanin biosynthesis have also been identified in multiple plant species. In maize, the bHLH TFs, R, B, Sn, and Hopi, regulate anthocyanin biosynthesis in specific tissues, including the aleurone layer, scutellum, pericarp, root, mesocotyl, leaf, and anther (Styles et al., 1973; Tonelli et al., 1991; Goff et al., 1992; Procissi et al., 1997; Petroni et al., 2000). In rice, a mutation in the bHLH domain of the RC protein is responsible for the white pericarp phenotype of the grain (Sweeney et al., 2006). Homologs of the maize R and B TFs controlling anthocyanin biosynthesis in specific tissues are also known in *Antirrhinum* (*Delila*) (Carpenter et al., 1991), petunia (*Jaf13*) (Quattrocchio et al., 1998), tomato (*AH*) (Qiu et al., 2016), and *Ipomoea purpurea* (*bHLH2*) (Park et al., 2007).

AP accumulation has frequently been found in many colored wheat tissues/organs, such as purple leaf blade, purple culm, purple glume, purple anther, purple pericarp, red coleoptile, red auricle, and red grain (Tereshchenko et al., 2013). These color traits have traditionally been used as visual markers for genetic analysis (Zeven, 1991). But in recent years there are increasing interests in developing the wheat cultivars with colored grains for manufacturing functional foods (Li et al., 2007; Khlestkina et al., 2011; Revanappa and Salimath, 2011). The molecular genetic basis controlling tissue/organ-specific accumulation of APs in wheat has been investigated by several studies. Two MYB TFs, R, and Rc, are found involved in the regulation of proanthocyanidin synthesis in wheat grains and coleoptiles (Himi et al., 2011; Himi and Taketa, 2015; Wang Y. Q. et al., 2016). Some insights have also been gained into the MYB and bHLH TFs controlling AP biosynthesis in purple pericarp, which is the main site of AP accumulation in purple wheat grains. Classic genetic analysis indicates that the homoeoallelic *Pp1* genes on the short arms of group 7 chromosomes and the *Pp3* gene on chromosome arm 2AL of common wheat control red pericarp (Knievel et al., 2009). Subsequent investigations suggest that *Pp1* may be orthologous to *ZmC1* of maize and *OsC1* of rice, which encode MYB-like TFs responsible for the activation of anthocyanin biosynthesis genes (Saitoh et al., 2004; Khlestkina, 2013). Recently, a genetic analysis proposed that *TaMYC1*, a putative wheat MYC TF gene, may be *Pp3*, based on co-location of *TaMYC1* with the *Pp3* gene conferring purple pericarp color and the transcript level of the dominant *TaMYC1* allele being much higher in purple pericarp tissues as a functional evidence (Shoeva et al., 2014). In our expression profile analysis, we also observed that the expression level of *TaMYC1* was higher in purple wheat grains than in white wheat grains (Liu et al., 2016). Nevertheless, there is still no strong molecular genetic evidence supporting the function of *TaMYC1* in regulating anthocyanin biosynthesis in purple wheat grains. Furthermore, it is not known if the *TaMYC1* alleles in purple and white pericarp tissues may differ in structure and function.

Based on the information above, the main objectives of this work were to examine if *TaMYC1* may regulate anthocyanin biosynthesis in purple pericarp and to characterize the *TaMYC1* alleles from purple- and white-grained wheat, respectively. By combining molecular and genetic investigations, we found that manipulation of *TaMYC1* expression directly affected anthocyanin biosynthesis. We isolated two *TaMYC1* alleles, *TaMYC1p* and *TaMYC1w*, from purple- and white-grained wheat cultivars, respectively, and found that their promoter regions differed substantially. These functional verification and association analysis data lead us to suggest that *TaMYC1* regulates anthocyanin biosynthesis in the purple pericarp tissues of common wheat, and that *TaMYC1p* represents a novel bHLH TF gene allele.

## Results

### Molecular characteristics of *TaMYC1*

Previous studies indicated that *TaMYC1* was located on 2AL (Shoeva et al., 2014; Liu et al., 2016). Based on the information reported in these studies, we designed further experiments to investigate the molecular characteristics of *TaMYC1*. The genomic and cDNA sequences of *TaMYC1* were isolated from the common wheat cultivars Gaoyuan 115 (purple-grained) and Opata (white-grained), respectively. The genomic region containing the *TaMYC1* open reading frame (ORF) was found to be 4,584 bp in both Gaoyuan 115 and Opata.

**Figure 1:**
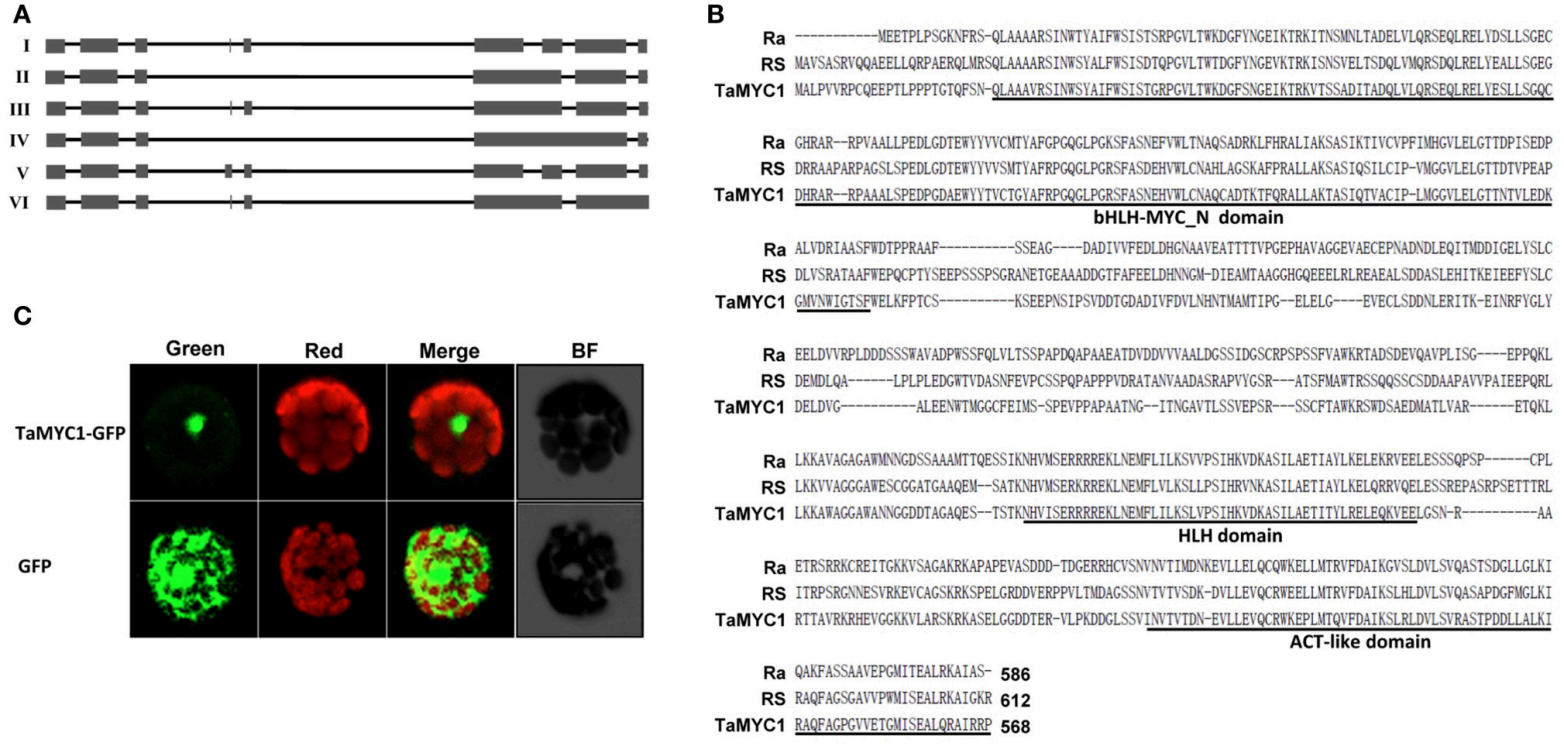
Molecular analysis of *TaMYC1* transcripts and deduced protein. **(A)** The six transcript isoforms (I–VI) identified for *TaMYC1*. The number of exons (represented by filled boxes) covered by the six isoforms varied from five (Isoform IV) to nine (Isoform V). **(B)** Amino acid sequence comparison between TaMYC1 (deduced from Isoform III) and two representative bHLH regulators of anthocyanin biosynthesis, RS from maize and Ra from rice. The three domains (bHLH-MYC_N domain, HLH domain and ACT-like domain) conserved among known bHLH TFs regulating anthocyanin biosynthesis are underlined. The GenBank accession numbers of the three sequences are NP_001106073 (RS), AAC49219 (Ra), or KX867111 (TaMYC1). **(C)** Localization of TaMYC1-GFP fusion protein in the nucleus of *Arabidopsis* protoplast. As a control, the free GFP protein was distributed throughout the cytoplasm of the protoplast. The images shown were taken under a confocal microscope using green (for GFP fluorescence) or red (for chlorophyll fluorescence) filters or under bright field (BF). The merged images depicted more clearly the relative positions of GFP and chlorophyll fluorescence in the photographed protoplasts.

Sequencing *TaMYC1* cDNAs amplified from Gaoyuan 115 grain tissues identified six different transcript isoforms (Isoforms I–VI, Figure 1A), with Isoform III accounting for ~86.6% of the total transcripts (Table S1). The ORF size of the six isoforms varied from 1,566 to 1,798 bp (Table S1), and the number of exons covered by them differed from five to nine (Figure 1A). The genomic ORF of *TaMYC1* in Opata was identical to that of Gaoyuan 115. The six transcript isoforms of *TaMYC1* were also found in the grain tissues of Opata, though in this cultivar the transcript level of *TaMYC1* was much lower (see below).

Conceptual translation of the six isoforms yielded polypeptides containing 407–580 amino acids (Table S1). However, only the deduced protein of Isoform III contained all three domains (bHLH-MYC_N, HLH, and ACT-like) shared by the MYC TFs previously found involved in controlling AP biosynthesis (Figure 1B, Figure S1). On the other hand, the translated product of Isoform I had 47 amino acids deleted in the HLH domain; a stretch of 24 residues were deleted in the bHLH-MYC_N domain in the polypeptide derived from Isoform II; the deduced product of Isoform IV had 24 amino acids deleted in the bHLH-MYC_N domain and an insertion of “TRTRTPPKSKRKEKKYstop” in the HLH domain; the deduced polypeptide of Isoform V had an insertion of “GAHACYLCRLNQ” in the ACT-like domain; an insertion of “VWEstop” in the ACT-like domain was found for the polypeptide translated from Isoform VI (Figure S1).

A TaMYC1 (Isoform III)-GFP fusion cistron, directed by the cauliflower mosaic virus 35S promoter, was constructed and transiently expressed in *Arabidopsis* protoplasts. The results showed that the TaMYC1-GFP fusion protein was located in the nucleus, whereas the control GFP protein was distributed throughout the cell (Figure 1C).

### Expression analysis of *TaMYC1*

**Figure 2:**
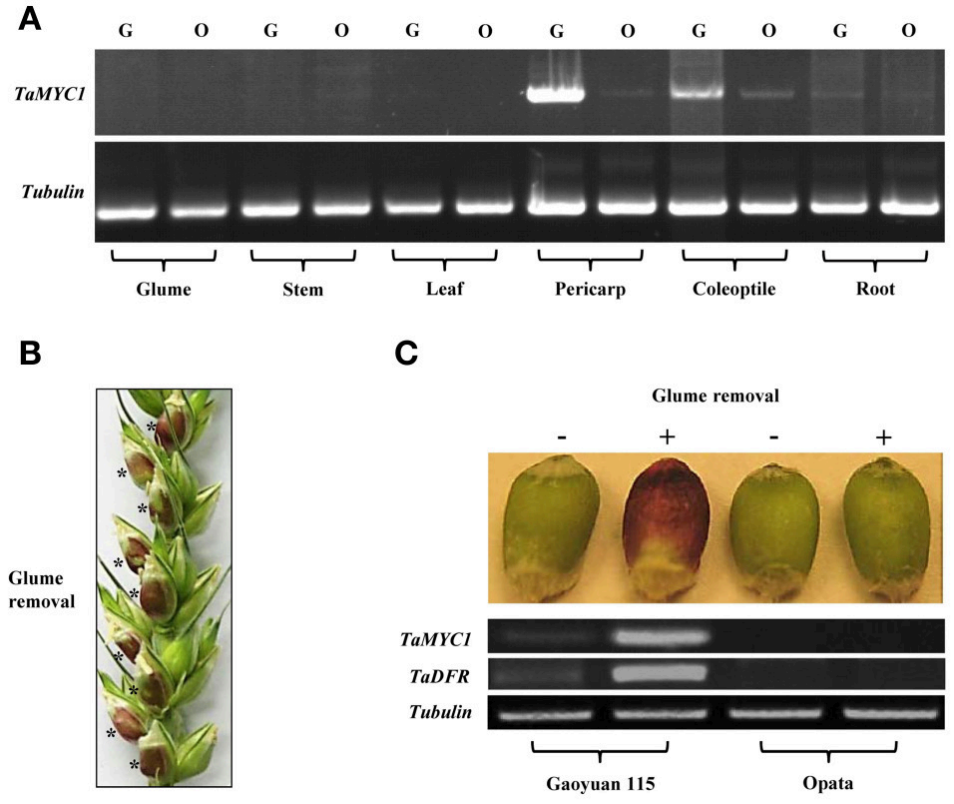
Transcriptional characteristics of *TaMYC1*. **(A)** Relative transcript levels of *TaMYC1* in the different organs/tissues (glume, stem, leaf, pericarp, coleoptile, and root) of Gaoyuan 115 (G) and Opata (O) as assessed using semi-quantitative RT-PCR. The amplification of wheat tubulin gene served as an internal control. **(B)** Artificial removal of outer and inner glumes induced purple AP accumulation in the developing grains of Gaoyuan 115. Glume removal was conducted at 14 days after flowering, with AP induction becoming visible in the grains (indicated by asterisks) 2 days after the treatment. **(C)** Relative transcript levels of *TaMYC1* and *TaDFR* in the grains of Gaoyuan 115 and Opata without (−) or with (+) glume removal treatment. The transcript levels were evaluated using semi-quantitative RT-PCR with the amplification of wheat tubulin gene as an internal control. The data displayed are representative of three separate tests.

The transcriptional behavior of *TaMYC1* in several different tissues of Gaoyuan 115 and Opata was investigated by semi-quantitative PCR with a pair of primers capable of recognizing all six transcript isoforms. As shown in Figure 2A, for both cultivars, the transcript level of *TaMYC1* was highest in pericarp tissues, intermediate in coleoptile and root tissues, and undetectable in leaf, stem, and glume tissues. But notably, *TaMYC1* transcripts were substantially more abundant in the pericarp, coleoptile, and root cells of Gaoyuan 115 than in those of Opata (Figure 2A).

Past studies have shown that artificial light exposure can stimulate AP accumulation in plant organs (Singh et al., 1999; Takos and Walker, 2006; Meng and Liu, 2015; Zhang et al., 2016). Therefore, we tested the effects of removing outer and inner glumes on AP accumulation and *TaMYC1* transcription in developing wheat grains. At 14 days after flowering (DAF), the glumes were carefully removed for one of the developing grains in a selected spikelet, with the glumes of the remaining grains retained as experimental controls (Figure 2B). Two days after the treatment, conspicuous purple AP accumulation was observed in the grains without glumes but not in the control ones with glume coverage in Gaoyuan 115 (Figure 2C). However, in Opata, no purple AP accumulation was found in either the grains with glume removal or the control ones (Figure 2C). The transcript level of *TaMYC1* was substantially up-regulated in the Gaoyuan 115 grains with glume removal, which was paralleled by a strong increase in the transcripts of *TaDFR*, an important anthocyanin biosynthesis gene coding for the enzyme dihydroflavonol 4-reductase (Figure 2C). Neither *TaMYC1* nor *TaDFR* were transcriptionally up-regulated in the grains of Opata irrespective of glume removal or retention (Figure 2C).

### Induction of purple AP accumulation by overexpression of *TaMYC1* and *ZmC1*

**Figure 3:**
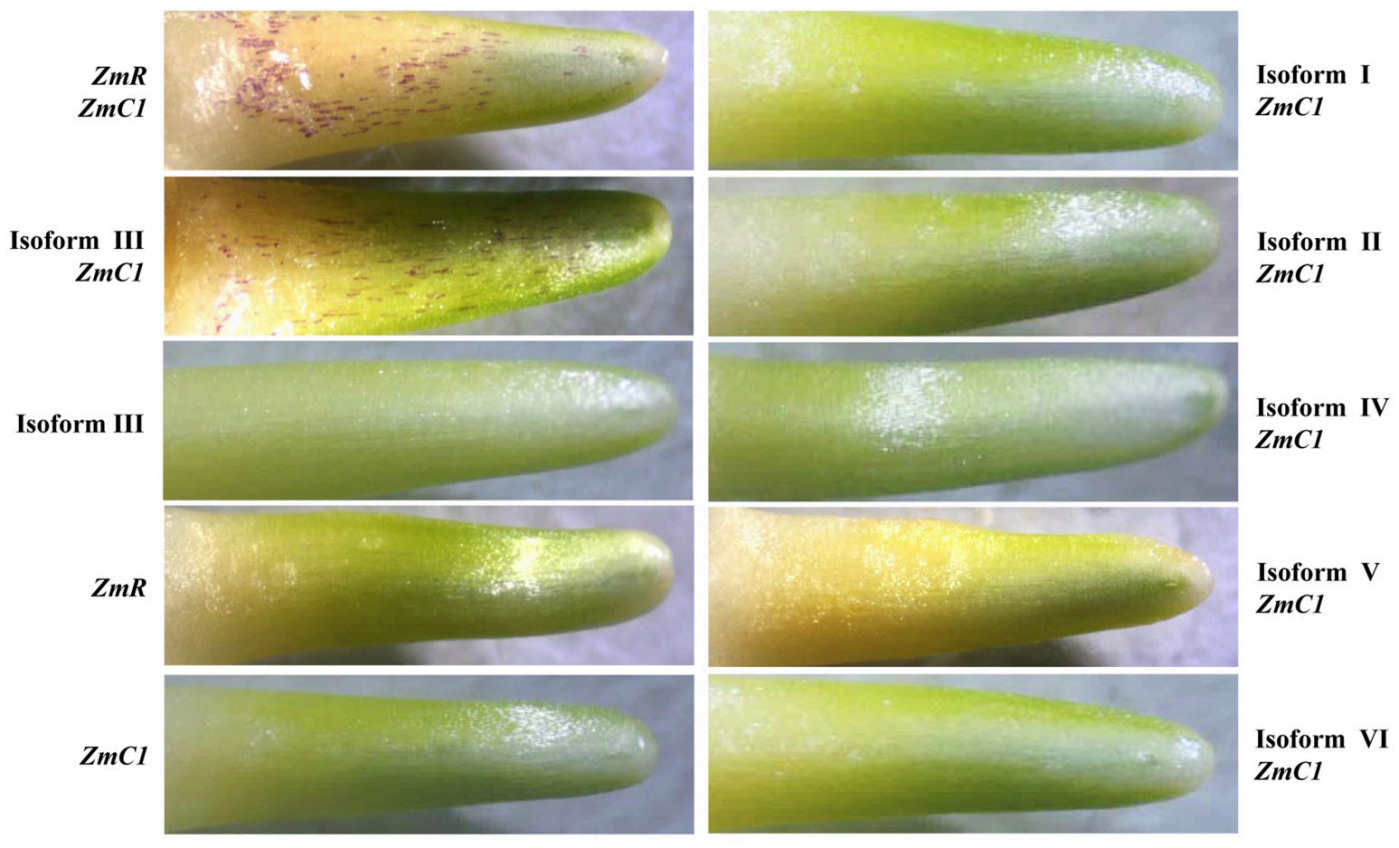
Induction of anthocyanin biosynthesis by *TaMYC1* in combination with *ZmC1*. The expression constructs of *ZmC1, ZmR*, and the six transcript isoforms of *TaMYC1* (Isoforms I–VI) were delivered into the coleoptile cells of the white-grain wheat Opata with particle bombardment in appropriate combinations or singularly. The presence of red colored cells in the bombarded coleoptiles indicates induction of anthocyanin biosynthesis. Of the six different types of transcripts of *TaMYC1*, only Isoform III induced anthocyanin biosynthesis in combination with *ZmC1*. The results shown are typical of three independent assays.

In previous research on maize anthocyanin regulators, coexpression of *ZmC1* (encoding a MYB TF) and *ZmR* (coding for a bHLH TF) was shown to be sufficient for inducing AP accumulation (Ludwig et al., 1989). Therefore, in this work, we tested if overexpression of *TaMYC1* and *ZmC1* may confer anthocyanin biosynthesis. The six transcript isoforms of *TaMYC1* was individually coexpressed with *ZmC1* in wheat coleoptile cells via particle bombardment mediated gene transfer (see Methods). As anticipated, simultaneous expression of *ZmC1* and *ZmR* conferred strong AP accumulation in the bombarded cells (Figure 3). Among the six transcript isoforms of *TaMYC1*, only Isoform III induced AP accumulation when coexpressed with *ZmC1* (Figure 3). On the other hand, expression of *ZmC1, ZmR*, or Isoform III alone failed to induce AP accumulation in the bombarded cells (Figure 3).

### Silencing *TaMYC1* expression inhibited purple AP accumulation in gaoyuan 115 grains

From the data present above, it became necessary to investigate if decreasing the expression of *TaMYC1* may reduce AP accumulation in purple-grained wheat. A virus induced gene silencing (VIGS) approach mediated by barley stripe mosaic virus (BSMV) was adopted for decreasing *TaMYC1* expression, because BSMV-VIGS has frequently been employed for functional studies of wheat genes (Wang et al., 2011; Zhou et al., 2011). Three recombinant BSMVs, including BSMV:GFP, BSMV:PDSas and BSMV:TaMYC1as, were used in this experiment. BSMV:GFP, expressing the green fluorescence protein (GFP) (Wang et al., 2011; Zhou et al., 2011), was used to monitor virus spread in the inoculated wheat plants. BSMV:PDSas, silencing wheat phytoene desaturase (PDS) gene and resulting photo bleaching (Scofield et al., 2005; Puri et al., 2007), provided a visual indication of positive gene silencing. BSMV:TaMYC1as was prepared in this work to silence *TaMYC1* expression in developing wheat grains.

**Figure 4:**
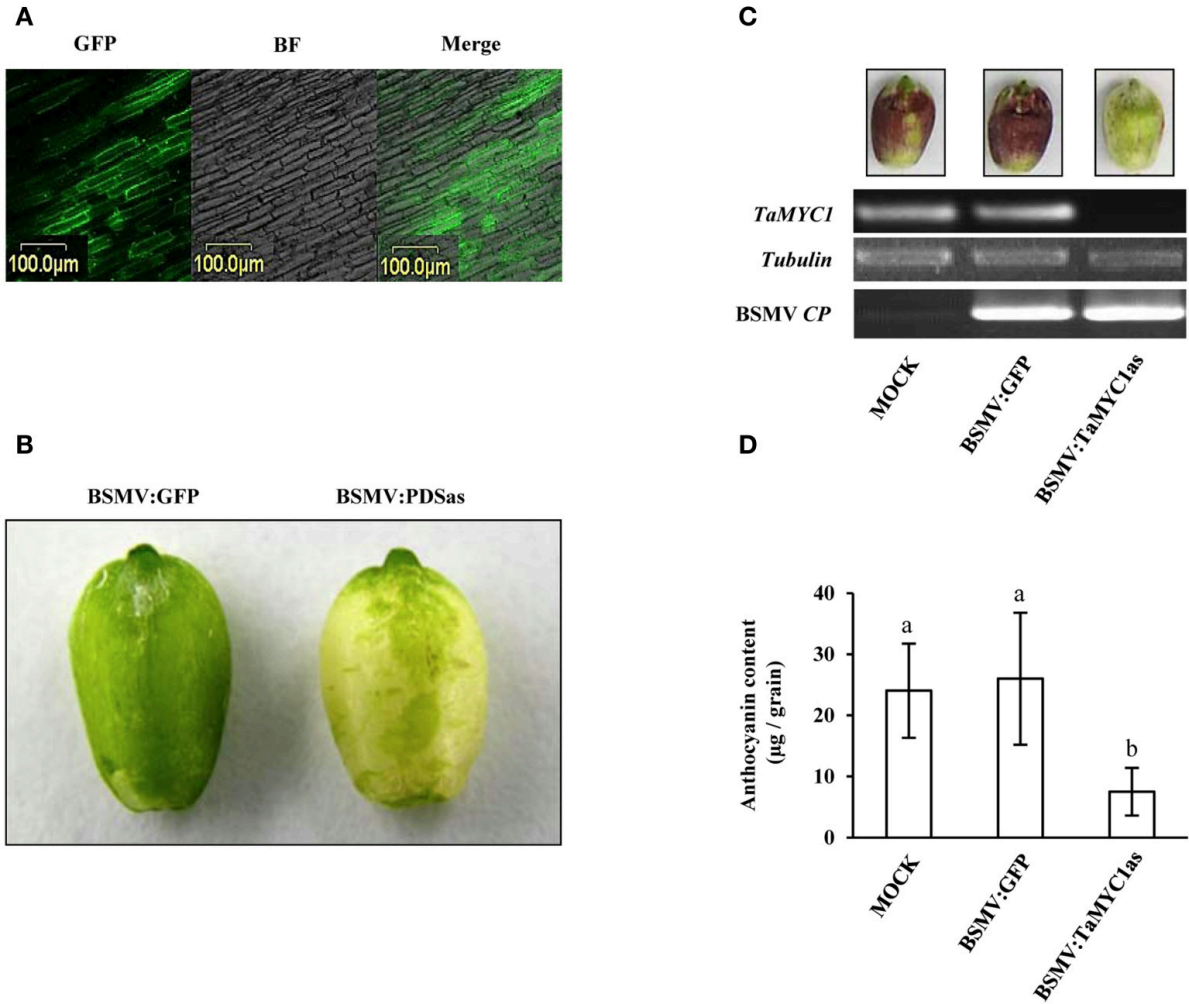
Analysis of the function of *TaMYC1* in regulating anthocyanin biosynthesis using virus induced gene silencing. Three recombinant barley stripe mosaic viruses (BSMV:GFP, BSMV:PDSas, and BSMV:TaMYC1as) were used in this experiment. Wheat plants (cv Gaoyuan 115) were inoculated with the three viruses, respectively. The developing grains were used for the experiment 14 days after the flowering. **(A)** GFP fluorescence was detected in the developing grains of the plants infected by BSMV:GFP. The images shown were taken under a confocal microscope in GFP channel and bright field (BF), respectively, followed by merging. **(B)** Photo bleaching was observed in the developing grains of the plants infected by BSMV:PDSas because of silencing the expression of phytoene desaturase gene. The bleaching phenotype did not occur in the developing grains of the plants infected by BSMV:GFP. **(C)** Evaluation of the relative transcript levels of *TaMYC1* and BSMV *CP* gene in the developing grains of mock controls and the plants infected by BSMV:GFP or BSMV:PDSas. The grains were subjected to glume removal, and at 2 days after the treatment, they were collected for this analysis. Purple anthocyanin pigments were induced in the grains of mock controls and the plants infected by BSMV:GFP but not those infected by BSMV:TaMYC1as. Consistent with this finding, *TaMYC1* transcripts accumulated in the grains of mock controls and the plants infected by BSMV:GFP, but were undetectable by RT-PCR in the grains of the plants infected by BSMV:TaMYC1as. Successful infection of the developing grains by BSMV:GFP or BSMV:PDSas was confirmed by positive detection of viral *CP* transcripts. The results depicted are representative of three independent experiments. **(D)** Comparison of anthocyanin contents among the developing grains of mock controls and the plants infected by BSMV:GFP or BSMV:TaMYC1as. The three sources of grains, as shown in **(C)**, were individually assayed for anthocyanin content, with the averaged values (means ± SE, *n* = 20) being compared statistically. The means marked by different letters are statistically significant (*P* < 0.05). The data shown were reproducible in another two separate determinations.

The three viruses were each introduced into Gaoyuan 115 plants through transcript inoculation of young flag leaves, with the buffer inoculated plants as mock controls (see Methods). The treated plants flowered at ~2.5 weeks post-inoculation. At 14 DAF, GFP fluorescence was detected in the pericarp cells of the plants inoculated by BSMV:GFP (Figure 4A). Photo bleaching was observed on the grains collected from the plants inoculated with BSMV:PDSas but not on those from the mock controls (Figure 4B). The grains in the BSMV:TaMYC1as plants did not show photo bleaching either. After glume removal and exposure to light, purple anthocyanins were strongly accumulated in the grains of mock controls and the plants infected by BSMV:GFP but not in those infected by BSMV:TaMYC1as (Figure 4C). In agreement this finding, *TaMYC1* transcripts were found in the grains of mock controls and the plants infected by BSMV:GFP, but not detected by RT-PCR in the grains of the plants infected by BSMV:TaMYC1as (Figure 4C). Lastly, successful infection of the developing grains by BSMV:GFP or BSMV:PDSas was confirmed by positive detection of viral *CP* transcripts (Figure 4C). Quantitative measurement showed that the mean anthocyanin content of BSMV:TaMYC1as infected grains was reduced by 69.2% relative to that of BSMV:GFP infected grains, while there was no significant difference in this parameter between BSMV:GFP infected grains and those of mock controls (Figure 4D, Table S2).

### Sequence analysis of *TaMYC1P* and *TaMYC1W* alleles

**Figure 5:**
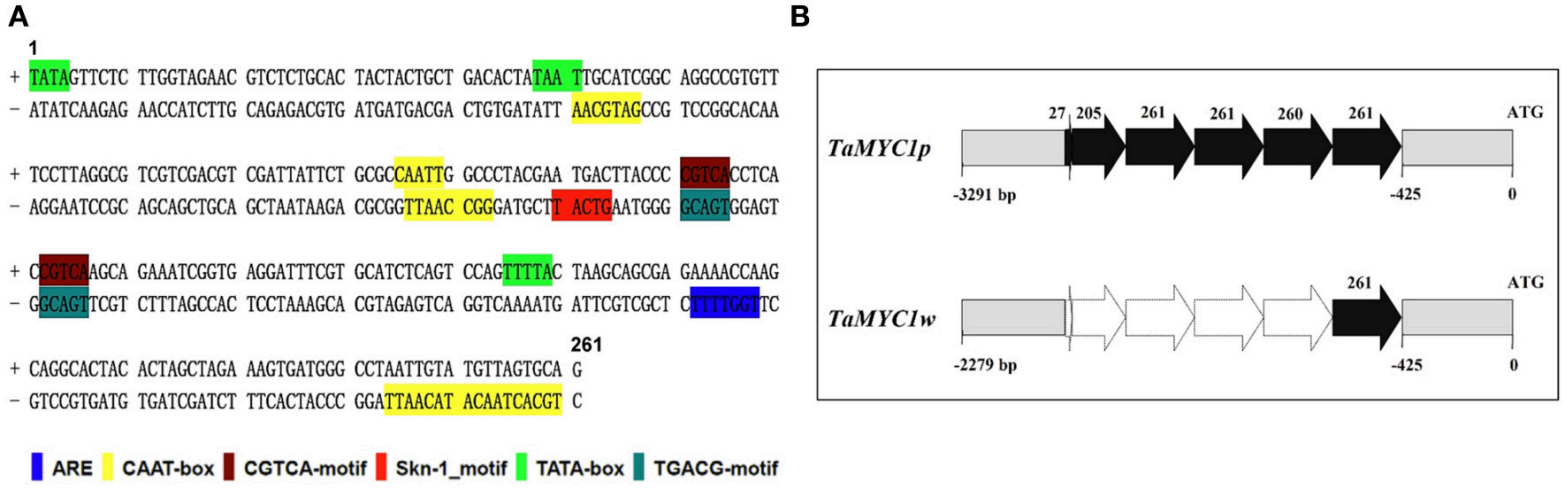
Bioinformatic analysis of a putative, 261 nt *cis*-regulatory element in the promoter region of *TaMYC1*. **(A)** Presence of multiple *cis*-acting regulatory motifs (boxes) in the 261 nt element as predicted using the PlantCARE software. **(B)** A diagram illustrating copy number difference of the 261 nt element in the promoter regions of two *TaMYC1* alleles (*TaMYC1p* and *TaMYC1w*) isolated from purple- and white-grained wheat cultivars, respectively. The 261 nt element had three perfect (261 nts), one nearly intact (260 nts) and two partial (27 or 205 nts) copies in the promoter of *TaMYC1p*, but was present only once (261 nts) in the corresponding region of *TaMYC1w*. ATG indicates the start codon of the coding sequence.

The 5′ proximal region of *TaMYC1* was isolated from both Gaoyuan 115 and Opata. It was found to be 3,291 bp in Gaoyuan 115 and 2,279 bp in Opata. Analysis of the resultant sequences identified a repeated sequence element of 261 nucleotides (nts), which had three prefect (261 nts), one nearly intact (260 nts), and two incomplete copies (27 and 205 nts, respectively) in the promoter of the *TaMYC1* allele in Gaoyuan 115 (designated as *TaMYC1p*), but was present only once (261 nts) in the corresponding region of the *TaMYC1* allele in Opata (designated as *TaMYC1w*) (Figures 5A,B). By analysis with the software PlantCARE, this 261 nt element was found to contain 16 copies of previously identified *cis*-acting regulatory motifs (boxes), including one ARE motif, eight CAAT boxes, two CGTCA motifs, one Skn-1 motif, three TATA boxes, and one TGACG motif (Figure 5A, Table S3). Interestingly, this putative, compound *cis*-acting regulatory element had not been identified and characterized by past studies. Searching public nucleic acid and genomic databases indicated that it was present exclusively in the promoter region of predicted bHLH TF genes in Triticeae species (Table S4). However, in these predicted bHLH TF genes, the copy number of the 261 nt element was generally ≤ 2 (Table S4), which was much fewer than that found in the promoter region of *TaMYC1p* (Figure 5). Together, the results above indicated that *TaMYC1p* and *TaMYC1w* alleles differed strongly in the promoter region, despite that they had an identical coding sequence.

### Segregation of pericarp colors and *TaMYC1* alleles in a RIL population

A polymorphic PCR marker, *Xtamyc1*, was designed based on nucleotide sequence difference between the promoter regions of *TaMYC1p* and *TaMYC1w*. The amplicons yielded by *Xtamyc1* were either 2,163 bp (for *TaMYC1p*) or 1,151 bp (for *TaMYC1w*) (Figure S2). A total of 185 RILs developed using Gaoyuan 115 and Opata as parents were examined for pericarp colors. The numbers of RILs with purple or white pericarp were 55 and 130, respectively (Table 1). When screened using *Xtamyc1*, the 55 purple-grained RILs all carried *TaMYC1p*, but for the 130 white-grained RILs, 90 had *TaMYC1w*, and the remaining 40 carried *TaMYC1p* (Table 1).

**Table 1:** Segregation of pericarp colors and *TaMYC1* alleles in the RILs derived from the cross between Gaoyuan 115 and Opata.

**Pericarp color**	***TaMYC1*** **allele**		**Total**
	***TaMYC1p***	***TaMYC1w***	
Purple	55	0	55
White	40	90	130
Total	95	90	185

### Association between purple pericarp color and *TaMYC1P* allele in diploid, tetraploid, and common wheat germplasm materials

It is well-known that common wheat was evolved through two polyploidization events (Nesbitt, 2001). The first one involved the diploid wheat *T. urartu* (AA, 2*n* = 2x = 14) and an *Aegilops* species (carrying the B genome), and formed tetraploid wheat (AABB, 2*n* = 4x = 28). The second one occurred between tetraploid wheat and the diploid goatgrass *Ae. tauschii* (DD, 2*n* = 2x = 14), and resulted in common wheat. Consequently, the A and D subgenomes of common wheat were donated by *T. urartu* and *Ae. tauschii*, respectively, and tetraploid wheat played an essential role in the evolution of common wheat. Both *T. urartu* and tetraploid wheat have many genetically different forms, which are important germplasm materials for wheat genetic, evolutionary and breeding studies (Salamini et al., 2002). Based on the above information, we investigated the allelic status of *TaMYC1* in multiple lines of *T. urartu*, tetraploid wheat and common wheat using *Xtamyc1* marker in order to explore potential association between purple pericarp color and *TaMYC1p* allele in wheat germplasm materials (Table 2). Because it has been suggested that purple pericarp in common wheat may be originally derived from tetraploid wheat (Zeven, 1991), we included three species of tetraploid wheat and relatively more purple-grained tetraploid wheat lines in this analysis (Table 2). The 98 *T. urartu* accessions all had white pericarp, and they were all found to carry *TaMYC1w*. Among the 256 durum wheat lines, 236 had purple pericarp and carried *TaMYC1p*; the remaining 20 lines had white pericarp and possessed *TaMYC1w*. For another two tetraploid wheat species (*T. turgidum* ssp. *turgidum* and *T. turgidum* ssp. *polonicum*), the 12 purple-grained lines were all found to host *TaMYC1p*, whereas the 20 white-grained lines all carried *TaMYC1w*. Of the 102 common wheat lines, 14 had purple pericarp and possessed *TaMYC1p*; 88 were white-grained with only *TaMYC1w* detected in them (Table 2).

**Table 2:** Association between pericarp colors and *TaMYC1* alleles in diploid, tetraploid, and hexaploid wheat germplasm lines.

**Genome**	**Species**	**Pericarp color**	**Number of lines**	***TaMYC1*** **allele**	
				***TaMYC1p***	***TaMYC1w***
AA (Diploid)	*T. urartu*	White	98	0	98
AABB (Tetraploid)	*T. turgidum* ssp. *durum*	Purple	236	236	0
		White	20	0	20
	*T. turgidum* ssp. *polonicum*	Purple	2	2	0
		White	3	0	3
	*T. turgidum* ssp. *turgidum*	Purple	10	10	0
		White	17	0	17
AABBDD (Hexaploid)	*T. aestivum*	White	88	0	88
		Purple	14	14	0
Total			488	262	226

## Discussion

In this work, we investigated the function of *TaMYC1* in regulating anthocyanin biosynthesis, and isolated two different alleles of *TaMYC1* from purple- and white-grained wheat, respectively. The new insights obtained and their implications for further research are discussed below.

### *TaMYC1* regulates anthocyanin biosynthesis

Prior to this work, *TaMYC1* had been implicated in the control of purple pericarp in wheat (Shoeva et al., 2014; Liu et al., 2016), although no strong molecular evidence was available for its function in anthocyanin biosynthesis. Here, we obtained complementary molecular and genetic evidence for the regulation of anthocyanin biosynthesis by *TaMYC1*. First, *TaMYC1* encoded a bHLH protein that was targeted to plant nucleus, and homologous to the bHLH TFs (e.g., Ra and RS) known to be involved in the control of anthocyanin biosynthesis (Figure 1). Second, the transcript level of *TaMYC1* was substantially higher in purple pericarp tissues relative to white pericarp tissues (Figure 2A), which consistent with earlier studies (Shoeva et al., 2014; Liu et al., 2016). Our result was based on the transcript comparison of *TaMYC1* between the white grain cultivar Opata and the purple grain cultivar Gy115, while the earlier study was carried out in NILs (Liu et al., 2016). Furthermore, our glume removal experiment showed clearly a parallel between light enhanced transcription of *TaMYC1* and increased accumulation of purple APs in the pericarp tissues of purple-grained, but not white-grained, wheat (Figure 2B). Third, simultaneous overexpression of *TaMYC1* and *ZmC1* mimicked the effects of coexpression of *ZmR* and *ZmC1*, both of which are validated regulators of anthocyanin biosynthesis (Pazares et al., 1987; Ludwig et al., 1989), on the induction of purple AP accumulation in wheat coleoptile cells (Figure 3). Lastly, decreasing the transcript level of *TaMYC1* through VIGS inhibited the accumulation of APs, and dramatically reduced anthocyanin content in a purple-grained wheat cultivar (Figure 4). Collectively, the above evidence suggests that (1) *TaMYC1* is a functional analog of *ZmR* in regulating anthocyanin biosynthesis in plant cells, (2) *TaMYC1*, with a relatively higher transcript level in the purple pericarp tissues, is necessary for AP accumulation in purple-grained wheat, and (3) The lower transcript level of *TaMYC1* in the white pericarp tissues may contribute to the lack of AP accumulation in white-grained wheat. Nevertheless, the higher expression level of *TaMYC1* is unlikely the solely promoter of anthocyanin accumulation in the purple pericarp tissues of Gaoyuan 115; the lower expression level of *TaMYC1* may not be the only reason for the lack of anthocyanin accumulation in the white pericarp tissues of Opata. By analogous to previous studies (Khlestkina, 2013; Tereshchenko et al., 2013; Shoeva et al., 2014; Liu et al., 2016), a MYB TF gene is required for the promotion of anthocyanin accumulation by *TaMYC1* (see also below). This MYB TF gene should be functional in Gaoyuan 115 grains. Further study is required to isolate this gene and to examine its functional interaction with *TaMYC1*.

Although *TaMYC1* had multiple transcript isoforms (Figure 1A), the dominant isoform (Isoform III) accounted for more than 80% of the transcripts, and encoded a functional bHLH protein capable of promoting AP accumulation in wheat coleoptile cells with the aid of *ZmC1* (Figure 3). Unlike Isoform III, the other minor transcript isoforms could not cause AP accumulation when coexpressed with *ZmC1* (Figure 3). These results, plus the observation that an identical set of *TaMYC1* transcript isoforms was present in both purple and white pericarp tissues, indicate that the minor transcript isoforms may not play a significant role in the regulation of anthocyanin biosynthesis by *TaMYC1*. However, more efforts are needed to investigate if there might be additional and functional transcript isoforms of *TaMYC1* in the pericarp tissues.

It is interesting to note that a low level of *TaMYC1* transcripts was also present in the root tissues of Gaoyuan 115 and Opata (Figure 2A), although no purple anthocyanin pigments were visible in this organ. One possibility is that *TaMYC1* may be involved in the activation of flavonoid biosynthesis pathway in the roots, but the function of this pathway does not result in purple anthocyanin pigments in the root tissues owing to the lack of other gene(s). In line with this possibility, previous studies have shown that flavonoid biosynthesis pathway is active, and various flavonoid compounds are accumulated, in the roots of many plant species (Buer et al., 2006; Hernández-Mata et al., 2010; Wang H. et al., 2016).

### *TaMYC1p* represents a novel BHLH TF gene allele

Aside from confirming the function of *TaMYC1* in regulating anthocyanin biosynthesis, this work identified two distinct alleles of *TaMYC1* (i.e., *TaMYC1p* and *TaMYC1w*) from purple- and white-grained wheat, respectively. Interestingly, among the RILs segregating for pericarp color, purple pericarp co-segregated with *TaMYC1p*, but *TaMYC1p* was not strictly linked with purple pericarp (Table 1). This indicates that in the examined RIL population *TaMYC1p* is necessary but not sufficient for conferring purple pericarp. This may not be surprising considering that multiple TF genes (e.g., MYB and bHLH TF genes) have been found to regulate anthocyanin biosynthesis in plants (Zhang et al., 2014). Additionally, molecular variations in the structural genes of anthocyanin biosynthesis can also affect AP accumulation in plant organs (Kim et al., 2009; Ho and Smith, 2016). It is possible that the white-grained parent (i.e., Opata) lacks not only a functional *TaMYC1* allele but also additional genetic determinant(s) required for AP accumulation (e.g., a MYB TF gene). In this context, the RILs carrying *TaMYC1p* but with different pericarp colors are useful for identifying the additional genetic determinant(s) functioning in AP accumulation in further research.

*TaMYC1p* and *TaMYC1w* differed clearly in the promoter region with respect to the copy number of the 261 nt element (Figure 5). This putative, complex *cis*-acting regulatory element was present in multiple copies (three perfect and three partial) in the promoter region of *TaMYC1p* but only once in that of *TaMYC1w*. The homologs of the 261 nt element existed in only Triticeae species (barley, wheat, and related species), and were present exclusively in the promoter region of predicted bHLH TF genes (Table S4). Moreover, the homologous elements were present generally in low copy numbers (≤ 2) in the promoter region of these bHLH TF genes (Table S4). Thus, presence of multiple intact copies (≥3) of the 261 nt element in the promoter region was not found in any of the previously identified genes involved in the regulation of anthocyanin biosynthesis; it is unique for *TaMYC1p*, and makes this allele a novel genetic variant capable of enhancing AP accumulation in the purple pericarp of wheat. Since *TaMYC1p* and *TaMYC1w* did not differ in the coding sequence, it is tempting to suggest that variation in the copy number of the 261 nt element in the promoter region may be responsible for the functional difference between *TaMYC1p* and *TaMYC1w* in regulating AP accumulation in the pericarp. We are now in the process of testing this possibility.

### Origin of *TaMYC1p* in wheat

Previous genetic studies have suggested that purple pericarp is absent in diploid wheat, and that this trait may have evolved in the tetraploid wheat populations (Zeven, 1991). In this work, neither purple pericarp nor *TaMYC1p* allele were observed in the diploid wheat *T. urartu*, but purple pericarp was readily found in three species of tetraploid wheat examined, and all of the examined varieties with purple pericarp harbored *TaMYC1p* (Table 2). Our findings are consistent with the suggestions made by past studies on purple pericarp in wheat and closely related species, and further point out that the evolution of purple pericarp in tetraploid wheat is caused by the differentiation of *TaMYC1p* allele. Remarkably, *TaMYC1p* was present in all 15 purple-grained common wheat lines examined in this work. This indicates that the purple pericarp and *TaMYC1p* allele in these lines may be originally derived from tetraploid wheat through interspecific hybridization. However, the number of purple-grained common wheat lines investigated in this work is limited. Further study, involving the analysis of more diverse purple-grained common wheat materials, is needed to verify the above observation and speculation.

In summary, we generated convincing evidence for the function of *TaMYC1* in regulating anthocyanin biosynthesis in the pericarp tissues of common wheat. The novel *TaMYC1* allele, *TaMYC1p*, is a necessary genetic determinant of purple pericarp in wheat. *TaMYC1* and its alleles may aid further studies on the molecular mechanisms underlying anthocyanin biosynthesis and genetic enhancement of AP accumulation in wheat grains.

## Materials and methods

### Plant materials

Two main sets of wheat materials were used in this work. The first set included the common wheat varieties Gaoyuan 115 and Opata and the 185 RILs (at F8 generation) derived from a cross between the two varieties (with Gaoyuan 115 as female parent). Gaoyuan 115 was a stable and homozygous cultivar with red coleoptile and purple grain (Liu et al., 2016). The RIL population segregating for pericarp colors was developed using the single seed descent method (Tee and Qualset, 1975). The second set contained the wheat germplasm materials used for investigating the association between pericarp colors and *TaMYC1* alleles. These lines, including 98 accessions of *T. urartu*, 256 accessions of *T. turgidum* ssp. *durum*, 5 accessions of *T. turgidum* ssp. *polonicum*, 27 accessions of *T. turgidum* ssp. *turgidum*, and 102 accessions of *T. aestivum* (Table S5), were obtained from the National Plant Germplasm System of the US Department of Agriculture (http://www.ars-grin.gov/) and the Chinese Crop Germplasm Resource Center located in Xining, China.

### Preparation of genomic DNA, total RNA, and cDNA samples

Genomic DNA was isolated from the desired wheat samples using 1 g of 10-day-old seedlings (Yan et al., 2002). The coleoptile, husk, root, leaf, stem, and pericarp samples were collected from wheat plants as described previously (Tereshchenko et al., 2013). Total RNA was extracted from ~0.5 g of desired wheat tissues using the Tiangen RNAprep Pure Plant Kit (Tiangen Company, Beijing, China). The synthesis of cDNA from total RNA was accomplished using the First Strand cDNA Synthesis Kit (Thermo-Fisher Scientific, Shanghai, China) following the manufacturer's instructions.

### PCR and semi-quantitative PCR

PCR was conducted using the high-fidelity Phusion DNA polymerase (Thermo-Fisher Scientific, Beijing, China) under the following conditions: 2 min of denaturation at 98°C; 35 cycles of 15 s at 98°C, 30 s at 61°C, and 30 s at 72°C; followed by a final extension of 5 min at 72°C. The PCR products were cloned into the pGEM-T Easy Vector plasmid (Promega Corporation, Madison, USA). The recombinant plasmids were then transformed into *Escherichia coli* DH5α cells, with the positive clones sequenced commercially (Huada Gene, Shenzheng, China). All primers used in this study are listed in Table S6.

The semi-quantitative RT-PCR experiments in this work were conducted following a previous publication (Zhou et al., 2011). The amplification of wheat tubulin gene transcripts was used to normalize the cDNA contents of various reverse transcription mixtures before PCR, and to monitor the kinetics of thermo-amplification during PCR. The reproducibility of the transcriptional patterns revealed by semi-quantitative PCR was tested by at least three independent assays.

### Glume removal treatment and response of *TaMYC1* transcription to light

Gaoyuan 115 and Opata plants were grown in a greenhouse at 25°C (day)/20°C (night), with a photoperiod of 16 h light/8 h dark. At 14 days after anthesis, both the outer and inner glumes were carefully removed from 9 to 10 grains using forceps, with the remaining grains in the same spike untreated as controls. Afterwards, the plants were maintained under the same growth conditions. At 2 days after glume removal, the light exposed grains and the controls were photographed, and then used for investigating the transcriptional response of *TaMYC1* to light with semi-quantitative RT-PCR as described above.

### Transient expression experiments

For investigating the nuclear localization of TaMYC1, an expression construct (p35S-TaMYC1-GFP) was prepared by cloning TaMYC1 coding region upstream of that of GFP in the plasmid p35S-GFP (Liu et al., 2006). The two constructs (p35S-TaMYC1-GFP and p35S-GFP) were each delivered into *Arabidopsis* protoplasts using polyethylene glycol as reported previously (Liu et al., 2006). After 18 h of culture at 25°C, the protoplasts were examined under a Leica TCS SP2 confocal laser scanning microscope (Leica Microsystems, Heidelberg, Germany).

To test if *TaMYC1* may promote anthocyanin biosynthesis in the presence of *ZmR*, the six transcript isoforms (I–VI) of *TaMYC1* and the coding sequences of *ZmR* and *ZmC1* were each cloned downstream of the ubiquitin gene promoter in the plasmid vector pBRACT214 (Soltész and Vágújfalvi, 2013), resulting in the expression constructs pUbi-TaMYC1-I, pUbi-TaMYC1-II, pUbi-TaMYC1-III, pUbi-TaMYC1-IV, pUbi-TaMYC1-V, pUbi-TaMYC1-VI, pUbi-ZmR, and pUbi-ZmC1. These constructs were introduced into the coleoptile cells of Opata in the desired combinations or individually using particle bombardment (Ahmed et al., 2003). The coleoptiles were examined for purple AP accumulation at 2 days after bombardment, with the photographs taken under a stereoscope (Leica Co., Oskar-Barnack-Straße, Germany).

### Knocking down *TaMYC1* transcript level by VIGS

Among the three recombinant BSMVs used in this work, BSMV:GFP and BSMV:PDSas were prepared previously (Wang et al., 2011; Zhou et al., 2011), whereas BSMV:TaMYC1as was newly constructed following the method detailed in our prior study (Wang et al., 2011). A 200 bp cDNA fragment of *TaMYC1* was obtained by RT-PCR using the oligo nucleotide primers containing *Nhe*I sites (Table S6). This fragment replaced GFP coding sequence in the BSMV plasmid RNAγ_gammab:GFP_, giving rise to RNAγ_gammab:TaMYC1as_. The combination of RNAγ_gammab:TaMYC1as_ with the RNAα and RNAβ clones of BSMV formed BSMV:TaMYC1as. *In vitro* transcripts were prepared for the RNAα, RNAβ, and RNAγ clones of the three BSMVs, and inoculated onto the immature flag leaves of Gaoyuan 115 (Wang et al., 2011). Twenty plants were inoculated for each recombinant virus, and the same number of plants was buffered inoculated as mock controls. The inoculated plants were grown under normal greenhouse conditions (see above), with the developing grains used for the experiment 14 days after flowering. BSMV spread in the developing grains of the plants inoculated with BSMV:GFP was checked by examining GFP fluorescence under confocal microscope (see above). The progress of gene silencing was monitored through observing photo bleaching in the developing grains infected by BSMV:PDSas.

For assessing the transcript levels of *TaMYC1* and BSMV *CP* and the effects of knocking down *TaMYC1* on anthocyanin accumulation, glume removal was conducted for 240 grains in the mock control plants (80) and those infected by BSMV:GFP (80) or BSMV:TaMYC1as (80). Two days after glume removal, the light exposed grains were collected for subsequent analysis. Evaluation of the transcript levels of *TaMYC1* and BSMV *CP* was carried out by semi-quantitative RT-PCR as outlined above. The anthocyanin content of each grain was measured using the method for determining the total monomeric anthocyanin pigment content of plant juices and derivative products (AOAC Official Method 2005.02). For either the mock controls or the plants infected BSMV:GFP or BSMV:TaMYC1as, the assay of anthocyanin content was performed using three separate sets of grains (with 20 grains in each set). Statistical analyses of the data were performed using the software package SPSS for Windows 17. The method was UNIANOVA, and The *POST-HOC* was DUNCAN ALPHA (0.05).

### Genotyping RILs with *Xtamyc1*

To distinguish *TaMYC1p* from *TaMYC1w*, the polymorphic PCR marker, *Xtamyc1*, was designed according to nucleotide sequence difference between the promoter regions of the two alleles. The primers of *Xtamyc1* are listed in Table S6. The amplicons produced by *Xtamyc1* were 2,163 bp for *TaMYC1p* and 1,151 bp for *TaMYC1w* (Figure S2). The templates for genotyping with *Xtamyc1* were the genomic DNA samples of Gaoyuan 115, Opata, and derivative RILs extracted as above. The PCR conditions were also described as above, except that the extension time was changed to 2 min.

### Bioinformatic analysis

The exons covered by the six transcript isoforms of *TaMYC1* were analyzed using the Gene Structure Display Server (http://gsds.cbi.pku.edu.cn/). Amino acid sequence alignment was generated using the Vector NTI 10 software (Thermo-Fisher Scientific, Waltham, MA). Tandem Repeats Finder (http://tandem.bu.edu/trf/trf.html) was used to identify repeats in the promoter region of *TaMYC1*. The *cis*-acting regulatory motifs (boxes) in the 261 nt element were predicted with the PlantCARE software (http://bioinformatics.psb.ugent.be/webtools/plantcare/html/). Finally, the oligonucleotide primers used in this study were designed with the aid of Primer 5 software (Premier Biosoft, Palo Alto, CA, USA).

## Additional information

Accession codes: The genomic sequences of TaMYC1 from Gaoyuan 115 and Opata had been deposited in GenBank with accession numbers KX867111 and KX867112, respectively. The sequences of the six transcript isoforms of *TaMYC1* from Gaoyuan 115 were also submitted to GenBank with the accession numbers being KY499898–KY499903.

## Author contributions

HZ, BL, and DW designed the research. YZ, XX, and SL performed the experiments. WC and BZ contributed reagents and greenhouse facility to the work. YZ, XX, SL, BL, DL, and HZ analyzed the data. BL, DW, HZ, and YZ wrote the paper. All authors read and approved the final manuscript.

### Conflict of interest statement

The authors declare that the research was conducted in the absence of any commercial or financial relationships that could be construed as a potential conflict of interest.
