# Supplementary material for: Allelic Variation and Transcriptional Isoforms of Wheat TaMYC1 Gene Regulating Anthocyanin Synthesis in Pericarp
Source: Front Plant Sci. 2017 Sep 21;8:1645. doi: 10.3389/fpls.2017.01645 (PMC5613136; doi:10.3389/fpls.2017.01645)
Supplement: Supplementary file 1 [file Presentation1.PDF]

Allelic variation and transcriptional isoforms of wheat *TaMYC1* gene regulating anthocyanin synthesis in pericarp

Yuan Zong[1,2,*], Xin-yuan Xi[2,3,*], Shi-ming Li[2,*], Wen-jie Chen[2], Bo Zhang[2], Deng-cai Liu[4], Bao-long Liu[2], Daowen Wang[5] & Huai-gang Zhang[1,2]

[1]State Key Laboratory of Plateau Ecology and Agriculture, Qinghai University, Xining, Qinghai 800010, China. [2]Key Laboratory of Adaptation and Evolution of Plateau Biota, Northwest Institute of Plateau Biology, Chinese Academy of Sciences, Xining, Qinghai 810008, China. [3]University of Chinese Academy of Sciences, Beijing 100049, China. [4]Triticeae Research Institute, Sichuan Agricultural University, Chengdu, Sichuan 611130, China. [5]State Key Laboratory of Plant Cell and Chromosome Engineering, Institute of Genetics and Developmental Biology, Chinese Academy of Sciences, Beijing 100101, China.

[*]These authors contributed equally to this work.

[*]Corresponding authors: Bao-long Liu (blliu@nwipb.cas.cn), Daowen Wang (dwwang@genetics.ac.cn) & Huai-gang Zhang (hgzhang@nwipb.ac.cn).

FIGURE S1 | Development of the marker *Xtamyc1* for amplifying the two different alleles of *TaMYC1* (*TaMYC1p* and *TaMYC1w*). The fragments yielded by the marker were either 2163 or 1151 bp, which were indicative of *TaMYC1p* and *TaMYC1w,* respectively. The amplification results for two common wheat cultivars (Gaoyuan 115 and Opata) and three derivative recombinant inbred lines (RILs) were shown. The size (bp) of the DNA standards is shown on the left side of the graph.

FIGURE S2 | The alignment of amino acid sequences deduced from the six transcript isoforms of *TaMYC1*. The three domains (bHLH-MYC_N domain, HLH domain and ACT-like domain) conserved among known bHLH TFs regulating anthocyanin biosynthesis are underlined.

TABLE S1 | Molecular characteristics and relative abundance of the six transcript isoforms of *TaMYC1* found in the pericarp tissues of the purple-grained wheat Gaoyuan 115 [a,b].

TABLE S2 | The anthocyanin contents among the developing grains of mock controls and the plants infected by BSMV:GFP or BSMV:TaMYC1as [a].

TABLE S3 | The cis-acting regulatory motifs (boxes) predicted for the 261 nt element using the PlantCARE software.

TABLE S4 | Homologs of the 261 nt element found in the promoter region of predicted bHLH TF genes in Triticeae species [a].

TABLE S5 | The origin, pericarp phenotype and *TaMYC1* genotype of the wheat germplasm materials examined using *Xtamyc1* marker.

TABLE S6 | Oligonucleotide primers used in this work.

FIGURE S1

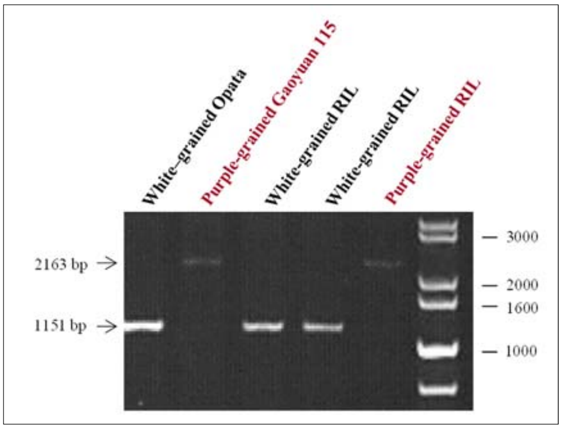
White-grained Opata
Purple-grained Gaoyuan 115
White-grained RIL
White-grained RIL
Purple-grained RIL
2163 bp
1151 bp
3000
2000
1600
1000

FIGURE S2

```
                    1                                                                                                  100
   Isoform I     (1) MALPVVRPCQEEPTLPPPTGTQFSNQLAAAVRSINWSYAIFWSISTGRPGVLTWKDGFSNGEIKTRKVTSSADITADQLVLQRSEQLRELYESLLSGQCD
  Isoform II     (1) MALPVVRPCQEEPTLPPPTGTQFSNQLAAAVRSINWSYAIFWSISTGRPGVLTWKDGFSNGEIKTRKVTSSADITADQLVLQRSEQLRELYESLLSGQCD
 Isoform III     (1) MALPVVRPCQEEPTLPPPTGTQFSNQLAAAVRSINWSYAIFWSISTGRPGVLTWKDGFSNGEIKTRKVTSSADITADQLVLQRSEQLRELYESLLSGQCD
  Isoform IV     (1) MALPVVRPCQEEPTLPPPTGTQFSNQLAAAVRSINWSYAIFWSISTGRPGVLTWKDGFSNGEIKTRKVTSSADITADQLVLQRSEQLRELYESLLSGQCD
   Isoform V     (1) MALPVVRPCQEEPTLPPPTGTQFSNQLAAAVRSINWSYAIFWSISTGRPGVLTWKDGFSNGEIKTRKVTSSADITADQLVLQRSEQLRELYESLLSGQCD
  Isoform VI     (1) MALPVVRPCQEEPTLPPPTGTQFSNQLAAAVRSINWSYAIFWSISTGRPGVLTWKDGFSNGEIKTRKVTSSADITADQLVLQRSEQLRELYESLLSGQCD

                    101                                                                                                200
   Isoform I   (101) HRARRPAAALSPEDPGDAEWYYTVCTGYAFRPGQGLPGRSFASNEHVWLCNAQCADTKTFQRALLAK------------TASIQTVACIPLMGGVLELGT
  Isoform II   (101) HRARRPAAALSPEDPGDAEWYYTVCTGYAFRPGQGLPGRSFASNEHVWLCNAQCADTKTFQRALLAK---------------------------------
 Isoform III   (101) HRARRPAAALSPEDPGDAEWYYTVCTGYAFRPGQGLPGRSFASNEHVWLCNAQCADTKTFQRALLAK------------TASIQTVACIPLMGGVLELGT
  Isoform IV   (101) HRARRPAAALSPEDPGDAEWYYTVCTGYAFRPGQGLPGRSFASNEHVWLCNAQCADTKTFQRALLAK---------------------------------
   Isoform V   (101) HRARRPAAALSPEDPGDAEWYYTVCTGYAFRPGQGLPGRSFASNEHVWLCNAQCADTKTFQRALLAKGAHACYLCRLNQTASIQTVACIPLMGGVLELGT
  Isoform VI   (101) HRARRPAAALSPEDPGDAEWYYTVCTGYAFRPGQGLPGRSFASNEHVWLCNAQCADTKTFQRALLAK------------TASIQTVACIPLMGGVLELGT
                                                                    bHLH-MYC_N domain
                    201                                                                                                300
   Isoform I   (189) TNTVLEDKGMVNWIGTSFWELKFPTCSKSEEPNSIPSVDDTGDADIVFDVLNHNTMAMTIPGELELGEVECLSDDNLERITKEINRFYGLYDELDVGALE
  Isoform II   (168) ---VLEDKGMVNWIGTSFWELKFPTCSKSEEPNSIPSVDDTGDADIVFDVLNHNTMAMTIPGELELGEVECLSDDNLERITKEINRFYGLYDELDVGALE
 Isoform III   (189) TNTVLEDKGMVNWIGTSFWELKFPTCSKSEEPNSIPSVDDTGDADIVFDVLNHNTMAMTIPGELELGEVECLSDDNLERITKEINRFYGLYDELDVGALE
  Isoform IV   (168) ---VLEDKGMVNWIGTSFWELKFPTCSKSEEPNSIPSVDDTGDADIVFDVLNHNTMAMTIPGELELGEVECLSDDNLERITKEINRFYGLYDELDVGALE
   Isoform V   (201) TNTVLEDKGMVNWIGTSFWELKFPTCSKSEEPNSIPSVDDTGDADIVFDVLNHNTMAMTIPGELELGEVECLSDDNLERITKEINRFYGLYDELDVGALE
  Isoform VI   (189) TNTVLEDKGMVNWIGTSFWELKFPTCSKSEEPNSIPSVDDTGDADIVFDVLNHNTMAMTIPGELELGEVECLSDDNLERITKEINRFYGLYDELDVGALE

                    301                                                                                                400
   Isoform I   (289) ENWTMGGCFEIMSSPEVPPAPAATNGITN-----------------------------------------------GAWANNGGDDTAGAQESTSTKNHV
  Isoform II   (265) ENWTMGGCFEIMSSPEVPPAPAATNGITNGAVTLSSVEPSRSSCFTAWKRSWDSAEDMATLVARETQKLLKKAWAGGAWANNGGDDTAGAQESTSTKNHV
 Isoform III   (289) ENWTMGGCFEIMSSPEVPPAPAATNGITNGAVTLSSVEPSRSSCFTAWKRSWDSAEDMATLVARETQKLLKKAWAGGAWANNGGDDTAGAQESTSTKNHV
  Isoform IV   (265) ENWTMGGCFEIMSSPEVPPAPAATNGITNGAVTLSSVEPSRSSCFTAWKRSWDSAEDMATLVARETQKLLKKAWAGGAWANNGGDDTAGAQESTSTKNHV
   Isoform V   (301) ENWTMGGCFEIMSSPEVPPAPAATNGITNGAVTLSSVEPSRSSCFTAWKRSWDSAEDMATLVARETQKLLKKAWAGGAWANNGGDDTAGAQESTSTKNHV
  Isoform VI   (289) ENWTMGGCFEIMSSPEVPPAPAATNGITNGAVTLSSVEPSRSSCFTAWKRSWDSAEDMATLVARETQKLLKKAWAGGAWANNGGDDTAGAQESTSTKNHV

                    401                                                                                                500
   Isoform I   (342) ISERRRREKLNEMFLILKSLVPSIHKVDKASILAETITYLRELEQKVEELGSNRAARTTAVRKRHEVGGKKVLARSKRKASELGGDDTERVLPKDDGLSS
  Isoform II   (365) ISERRRREKLNEMFLILKSLVPSIHKVDKASILAETITYLRELEQKVEELGSNRAARTTAVRKRHEVGGKKVLARSKRKASELGGDDTERVLPKDDGLSS
 Isoform III   (389) ISERRRREKLNEMFLILKSLVPSIHKVDKASILAETITYLRELEQKVEELGSNRAARTTAVRKRHEVGGKKVLARSKRKASELGGDDTERVLPKDDGLSS
  Isoform IV   (365) ISERRRREKLNEMFLILKSLVPSIHKVTRTRTPPKSKRKEKKY---------------------------------------------------------
   Isoform V   (401) ISERRRREKLNEMFLILKSLVPSIHKVDKASILAETITYLRELEQKVEELGSNRAARTTAVRKRHEVGGKKVLARSKRKASELGGDDTERVLPKDDGLSS
  Isoform VI   (389) ISERRRREKLNEMFLILKSLVPSIHKVDKASILAETITYLRELEQKVEELGSNRAARTTAVRKRHEVGGKKVLARSKRKASELGGDDTERVLPKDDGLSS
                            bHLH-MYC_N domain
                    501                                                                            580
   Isoform I   (442) VINVTVTDNEVLLEVQCRWKEPLMTQVFDAIKSLRLDVLSVRASTPDDLLALKIRAQFAGPGVVETGMISEALQRAIRRP
  Isoform II   (465) VINVTVTDNEVLLEVQCRWKEPLMTQVFDAIKSLRLDVLSVRASTPDDLLALKIRAQFAGPGVVETGMISEALQRAIRRP
 Isoform III   (489) VINVTVTDNEVLLEVQCRWKEPLMTQVFDAIKSLRLDVLSVRASTPDDLLALKIRAQFAGPGVVETGMISEALQRAIRRP
  Isoform IV   (408) --------------------------------------------------------------------------------
   Isoform V   (501) VINVTVTDNEVLLEVQCRWKEPLMTQVFDAIKSLRLDVLSVRASTPDDLLALKIRAQFAGPGVVETGMISEALQRAIRRP
  Isoform VI   (489) VINVTVTDNEVLLEVQCRWKEPLMTQVFDAIKSLRLDVLSVRASTPDDLLALKIRAQVWE--------------------
                                                  ACT-like domain
```

TABLE S1 | Molecular characteristics and relative abundance of the six transcript isoforms of *TaMYC1* found in the pericarp tissues of the purple-grained wheat Gaoyuan 115 [a,b].

	**Isoform I**	**Isoform II**	**Isoform III**	**Isoform IV**	**Isoform V**	**Isoform VI**
Open reading frame (ORF, bp)	1566	1635	1707	1741	1743	1798
Relative abundance [a]	4 (2.0%)	15 (7.5%)	174 (86.6%)	2 (1.0%)	2 (1.0%)	4 (2.0%)
Polypeptide deduced (aa) [b]	521	544	568	407	580	548

[a] The relative abundance of the six isoforms was estimated by cloning the RT-PCR products, sequencing 201 randomly picked positive clones, and counting the number of clones carrying the cDNA of a given transcript isoform.

[b] Each polypeptide was deduced from the corresponding ORF through conceptual translation. The six polypeptides differed in the number of amino acids (aa) because of the presence of indels and/or premature stop codon in their ORF.

TABLE S2 | The anthocyanin contents among the developing grains of mock controls and the plants infected by BSMV:GFP or BSMV:TaMYC1as[a].

	Mean anthocyanin content (μg /grain)	Standard deviation
Mock	24.03	7.71
BSMV:GFP	25.99	10.81
BSMV:TaMYC1as	7.51	3.89

[a] The three sources of grains were individually assayed for anthocyanin content, with the averaged values (means ± SE, n = 20) being compared statistically. The means marked by different letters are statistically significant ($P < 0.05$). The data shown were reproducible in another two separate determinations.

TABLE S3 | The *cis*-acting regulatory motifs (boxes) predicted for the 261 nt element using the PlantCARE software.

**Motif (Box)**	**Position**	**Strand**	**Matrix score**	**Sequence**	**function**
TATA-box	1	+	4	TATA	core promoter element around -30 of transcription start
TATA-box	46	+	4	TATA	core promoter element around -30 of transcription start
CAAT-box	49	-	5	CAATT	common cis-acting element in promoter and enhancer regions
CAAT-box	50	-	4	CAAT	common cis-acting element in promoter and enhancer regions
CAAT-box	104	+	5	CCAAT	common cis-acting element in promoter and enhancer regions
CAAT-box	105	+	5	CAATT	common cis-acting element in promoter and enhancer regions
CAAT-box	106	-	5	CAATT	common cis-acting element in promoter and enhancer regions
CAAT-box	107	-	5	CCAAT	common cis-acting element in promoter and enhancer regions
Skn-1_motif	120	-	5	GTCAT	cis-acting regulatory element required for endosperm expression
CGTCA-motif	131	+	5	CGTCA	cis-acting regulatory element involved in the MeJA-responsiveness
TGACG-motif	131	-	5	TGACG	cis-acting regulatory element involved in the MeJA-responsiveness
CGTCA-motif	142	+	5	CGTCA	cis-acting regulatory element involved in the MeJA-responsiveness
TATA-box	185	+	5	TTTTA	core promoter element around -30 of transcription start
ARE	203	-	6	TGGTTT	cis-acting regulatory element essential for the anaerobic induction
CAAT-box	244	-	5	CAATT	common cis-acting element in promoter and enhancer regions
CAAT-box	245	-	4	CAAT	common cis-acting element in promoter and enhancer regions

TABLE S4 | Homologs of the 261 nt element found in the promoter region of predicted bHLH TF genes in Triticeae species [a].

**Triticeae species**	**Database**	**Accession**	**Copy number**	**Identity**	**Percentage of identity**	**E-value**	**Start**	**End**	**Predicted gene**
Hordeum vulgare	Assembly_WGSBarke	Barke_contig_2804331	1	98/125	78	1E-22	1077	1240	bHLH TF
Hordeum vulgare	Assembly_WGSBowman	Bowman_contig_857662	1	95/124	76	2E-19	5803	5455	bHLH TF
Hordeum vulgare	Assembly_WGSMorex	Morex_contig_1573231	1	205/260	78	7E-58	109	409	bHLH TF
Triticum monococcum	Monococcum v1	TGAC_WGS_monococcum_v1_ contig_911585	1	261/261	100	2E-130	318	578	bHLH TF
Triticum monococcum	Monococcum v1	TGAC_WGS_monococcum_v1_ contig_948401	1	237/290	82	7E-74	2582	2294	bHLH TF
Triticum urartu	Urartu v1	TGAC_WGS_urartu_v1_contig_ 1358943	1	261/261	100	2E-130	954	694	bHLH TF
Triticum urartu	Urartu v1	TGAC_WGS_urartu_v1_contig_ 190389	1	237/290	82	7E-74	1036	1324	bHLH TF
Aegilops speltoides	Speltoides v1	TGAC_WGS_speltoides_v1_contig _226068	1	239/266	90	4E-96	5992	6256	bHLH TF
Aegilops speltoides	Speltoides v1	TGAC_WGS_speltoides_v1_contig _308503	1	250/273	92	2E-107	862	1134	bHLH TF
Aegilops tauschii	Tauschii v1	TGAC_WGS_tauschii_v1_contig_ 160595	1	184/196	94	9E-79	172	367	bHLH TF
Aegilops tauschii	Tauschii v1	TGAC_WGS_tauschii_v1_contig_ 1766537	1	236/294	80	5E-69	2664	2372	bHLH TF
Aegilops sharonensis	Sharonensis v1	TSL_WGS_sharonensis_v1_contig _1105393	1	218/239	91	1E-89	995	765	bHLH TF
Aegilops sharonensis	Sharonensis v1	TSL_WGS_sharonensis_v1_contig _17653	2	237/290	82	7E-74	1031	725	bHLH TF
Aegilops sharonensis	Sharonensis v1	TSL_WGS_sharonensis_v1_contig _17653	2	244/270	90	3E-98	1780	1512	bHLH TF
Triticum turgidum ssp. *durum*	Durum Cappelli v1	TGAC_WGS_durum_v1_contig_ 189553	1	244/253	96	2E-113	5731	5983	bHLH TF
Triticum turgidum ssp. *durum*	Durum Cappelli v1	TGAC_WGS_durum_v1_contig_ 2615326	1	261/261	100	2E-130	800	540	bHLH TF
Triticum turgidum ssp. *durum*	Durum Cappelli v1	TGAC_WGS_durum_v1_contig_ 281988	1	241/268	90	1E-96	1610	1344	bHLH TF

Triticum turgidum ssp. *durum*	Durum Cappelli v1	TGAC_WGS_durum_v1_contig_41798	1	237/290	82	7E-74	915	627	bHLH TF
Triticum turgidum L. var durum	Durum Strongfield v1	TGAC_WGS_strongfield_v1_contig_494358	1	261/261	100	2E-130	2733	2993	bHLH TF
Triticum turgidum L. var durum	Durum Strongfield v1	TGAC_WGS_strongfield_v1_contig_499303	1	128/132	97	7E-55	3792	3923	bHLH TF
Triticum turgidum L. var durum	Durum Strongfield v1	TGAC_WGS_strongfield_v1_contig_621749	1	237/290	82	7E-74	886	598	bHLH TF
Triticum turgidum L. var durum	Durum Strongfield v1	TGAC_WGS_strongfield_v1_contig_700959	1	133/157	85	3E-40	157	1	bHLH TF
Triticum aestivum	wheat sequence survey chromosome 2AL	IWGSC_chr2AL_ab_k71_contigs_longerthan_200_6338698	1	261/261	100	2E-130	2829	3089	bHLH TF
Triticum aestivum	wheat sequence survey chromosome 2AL	IWGSC_chr2AL_ab_k71_contigs_longerthan_200_6437660	1	237/290	82	5E-74	4169	4457	bHLH TF
Triticum aestivum	wheat sequence survey chromosome 2BL	IWGSC_chr2BL_ab_k71_contigs_longerthan_200_8014948	1	240/268	90	1E-95	269	535	bHLH TF
Triticum aestivum	wheat sequence survey chromosome 2DL	IWGSC_chr2DL_ab_k71_contigs_longerthan_200_5437347	1	184/196	94	7E-79	422	227	bHLH TF
Triticum aestivum	wheat sequence survey chromosome 2DL	IWGSC_chr2DL_ab_k71_contigs_longerthan_200_7235598	1	171/211	81	6E-48	211	1	bHLH TF

[a] The homologous sequences of the 261 nt element were searched using BlastN in four public nucleic acid and genomic databases, i.e., NCBI (https://blast.ncbi.nlm.nih.gov/), JGI (https://phytozome.jgi.doe.gov/), UIGI (https://urgi.versailles.inra.fr/) and IPK (http://webblast.ipk-gatersleben.de/barley). The homologs of the 261 nt element were found in only Triticeae species (i.e., barley, wheat and related *Aegilops* species), but not in the remaining 52 plant species (including rice, maize and *Brachypodium distachyon*) in JGI, even though the homologous sequence of *TaMYC1* coding region could be detected in these plants. The homologs of the 261 nt element were present exclusively in the promoter region of predicted bHLH TF genes in the concerned Triticeae species, and their copy numbers varied from one to two. “Accession” means the contig name. “Identity” indicates number of identical matches/alignment length. “Percentage of identity” means percentage of identical matches. “Start” means the beginning position of matches in the contig, while “End” means the final position of the matches in the contig.

TABLE S5 | The origin, pericarp phenotype and *TaMYC1* genotype of the wheat germplasm materials examined using *Xtamyc1* marker.

Item	Taxon	Plant ID	Cultivar	Country	Pericarp phenotype	TaMYC1 genotype
1	Triticum uratu	Cltr17664	G3135	Lebanon,EI Beqaa	White pericarp	TaMYC1w
2	Triticum uratu	Cltr17666	G3162	Lebanon,EI Beqaa	White pericarp	TaMYC1w
3	Triticum uratu	Cltr17667	G1785	Turkey,mardin	White pericarp	TaMYC1w
4	Triticum uratu	Cltr17668	G1753	Armenia	White pericarp	TaMYC1w
5	Triticum uratu	PI427328	G2264	Irap,Arbil	White pericarp	TaMYC1w
6	Triticum uratu	PI428180	G1734	Armenia	White pericarp	TaMYC1w
7	Triticum uratu	PI428181	G1753	Armenia	White pericarp	TaMYC1w
8	Triticum uratu	PI428182	G1754	Armenia	White pericarp	TaMYC1w
9	Triticum uratu	PI428183	G1759	Armenia	White pericarp	TaMYC1w
10	Triticum uratu	PI428184	G1785	Turkey,Mardin	White pericarp	TaMYC1w
11	Triticum uratu	PI428185	G1786	Turkey,Mardin	White pericarp	TaMYC1w
12	Triticum uratu	PI428186	G1787	Turkey,Mardin	White pericarp	TaMYC1w
13	Triticum uratu	PI428189	G1791	Turkey,Mardin	White pericarp	TaMYC1w
14	Triticum uratu	PI428190	G1792	Turkey,Mardin	White pericarp	TaMYC1w
15	Triticum uratu	PI428191	G1794	Turkey,Mardin	White pericarp	TaMYC1w
16	Triticum uratu	PI428194	G1798	Turkey,Mardin	White pericarp	TaMYC1w
17	Triticum uratu	PI428195	G1802	Turkey,Mardin	White pericarp	TaMYC1w
18	Triticum uratu	PI428196	G1810	Turkey,Mardin	White pericarp	TaMYC1w
19	Triticum uratu	PI428197	G1811	Turkey,Mardin	White pericarp	TaMYC1w
20	Triticum uratu	PI428198	G1812	Turkey,Mardin	White pericarp	TaMYC1w
21	Triticum uratu	PI428199	G1813	Turkey,Mardin	White pericarp	TaMYC1w
22	Triticum uratu	PI428200	G1817	Turkey,Mardin	White pericarp	TaMYC1w

23	Triticum uratu	PI428201	G1818	Turkey,Mardin	White pericarp	TaMYC1w
24	Triticum uratu	PI428202	G1821	Turkey,Mardin	White pericarp	TaMYC1w
25	Triticum uratu	PI428203	G1822	Turkey,Mardin	White pericarp	TaMYC1w
26	Triticum uratu	PI428204	G1824	Turkey,Mardin	White pericarp	TaMYC1w
27	Triticum uratu	PI428206	G1826	Turkey,Mardin	White pericarp	TaMYC1w
28	Triticum uratu	PI428207	G1827	Turkey,Mardin	White pericarp	TaMYC1w
29	Triticum uratu	PI428208	G1828	Turkey,Mardin	White pericarp	TaMYC1w
30	Triticum uratu	PI428209	G1829	Turkey,Mardin	White pericarp	TaMYC1w
31	Triticum uratu	PI428210	G1830	Turkey,Mardin	White pericarp	TaMYC1w
32	Triticum uratu	PI428211	G1831	Turkey,Mardin	White pericarp	TaMYC1w
33	Triticum uratu	PI428212	G1832	Turkey,Mardin	White pericarp	TaMYC1w
34	Triticum uratu	PI428213	G1833	Turkey,Mardin	White pericarp	TaMYC1w
35	Triticum uratu	PI428214	G1834	Turkey,Mardin	White pericarp	TaMYC1w
36	Triticum uratu	PI428215	G1836	Turkey,Mardin	White pericarp	TaMYC1w
37	Triticum uratu	PI428216	G1839	Turkey,Mardin	White pericarp	TaMYC1w
38	Triticum uratu	PI428218	G1843	Turkey,Mardin	White pericarp	TaMYC1w
39	Triticum uratu	PI428219	G1844	Turkey,Mardin	White pericarp	TaMYC1w
40	Triticum uratu	PI428220	G1865	Turkey,Urfa	White pericarp	TaMYC1w
41	Triticum uratu	PI428221	G1868	Turkey,Urfa	White pericarp	TaMYC1w
42	Triticum uratu	PI428222	G1876	Turkey,Urfa	White pericarp	TaMYC1w
43	Triticum uratu	PI428223	G1895	Turkey,Urfa	White pericarp	TaMYC1w
44	Triticum uratu	PI428224	G1899	Turkey,Mardin	White pericarp	TaMYC1w
45	Triticum uratu	PI428225	G1903	Turkey,Mardin	White pericarp	TaMYC1w
46	Triticum uratu	PI428226	G1904	Turkey,Mardin	White pericarp	TaMYC1w
47	Triticum uratu	PI428227	G1905	Turkey,Mardin	White pericarp	TaMYC1w

48	Triticum uratu	PI428228	G1906	Turkey,Mardin	White pericarp	TaMYC1w
49	Triticum uratu	PI428229	G1908	Turkey,Mardin	White pericarp	TaMYC1w
50	Triticum uratu	PI428230	G1937	Turkey,Urfa	White pericarp	TaMYC1w
51	Triticum uratu	PI428231	G1938	Turkey,Urfa	White pericarp	TaMYC1w
52	Triticum uratu	PI428232	G1939	Turkey,Urfa	White pericarp	TaMYC1w
53	Triticum uratu	PI428233	G1940	Turkey,Urfa	White pericarp	TaMYC1w
54	Triticum uratu	PI428234	G1941	Turkey,Urfa	White pericarp	TaMYC1w
55	Triticum uratu	PI428236	G1944	Turkey,Urfa	White pericarp	TaMYC1w
56	Triticum uratu	PI428237	G1945	Turkey,Urfa	White pericarp	TaMYC1w
57	Triticum uratu	PI428238	G1947	Turkey,Urfa	White pericarp	TaMYC1w
58	Triticum uratu	PI428240	G1949	Turkey,Urfa	White pericarp	TaMYC1w
59	Triticum uratu	PI428241	G1951	Turkey,Urfa	White pericarp	TaMYC1w
60	Triticum uratu	PI428243	G1953	Turkey,Urfa	White pericarp	TaMYC1w
61	Triticum uratu	PI428244	G1954	Turkey,Urfa	White pericarp	TaMYC1w
62	Triticum uratu	PI428245	G1955	Turkey,Urfa	White pericarp	TaMYC1w
63	Triticum uratu	PI428246	G1956	Turkey,Urfa	White pericarp	TaMYC1w
64	Triticum uratu	PI428247	G1958	Turkey,Urfa	White pericarp	TaMYC1w
65	Triticum uratu	PI428248	G1961	Turkey,Urfa	White pericarp	TaMYC1w
66	Triticum uratu	PI428249	G1965	Turkey,Urfa	White pericarp	TaMYC1w
67	Triticum uratu	PI428250	G1966	Turkey,Urfa	White pericarp	TaMYC1w
68	Triticum uratu	PI428251	G1967	Turkey,Urfa	White pericarp	TaMYC1w
69	Triticum uratu	PI428252	G2035	Turkey,Urfa	White pericarp	TaMYC1w
70	Triticum uratu	PI428253	G2582	Iraq,Arbil	White pericarp	TaMYC1w
71	Triticum uratu	PI428254	G2194	Turkey,Mus	White pericarp	TaMYC1w
72	Triticum uratu	PI428255	G2949	Turkey,Mardin	White pericarp	TaMYC1w

73	Triticum uratu	PI428257	G2989	Armenia	White pericarp	TaMYC1w
74	Triticum uratu	PI428258	G2990	Armenia	White pericarp	TaMYC1w
75	Triticum uratu	PI428259	G2991	Armenia	White pericarp	TaMYC1w
76	Triticum uratu	PI428260	G3135	Lebanon,EI Beqaa	White pericarp	TaMYC1w
77	Triticum uratu	PI428261	G3140	Lebanon,EI Beqaa	White pericarp	TaMYC1w
78	Triticum uratu	PI428262	G3141	Lebanon,EI Beqaa	White pericarp	TaMYC1w
79	Triticum uratu	PI428263	G3142	Lebanon,EI Beqaa	White pericarp	TaMYC1w
80	Triticum uratu	PI428264	G3143	Lebanon,EI Beqaa	White pericarp	TaMYC1w
81	Triticum uratu	PI428265	G3144	Lebanon,EI Beqaa	White pericarp	TaMYC1w
82	Triticum uratu	PI428267	G3148	Lebanon,EI Beqaa	White pericarp	TaMYC1w
83	Triticum uratu	PI428268	G3150	Lebanon,EI Beqaa	White pericarp	TaMYC1w
84	Triticum uratu	PI428269	G3151	Lebanon,EI Beqaa	White pericarp	TaMYC1w
85	Triticum uratu	PI428270	G3152	Lebanon,EI Beqaa	White pericarp	TaMYC1w
86	Triticum uratu	PI428271	G3154	Lebanon,EI Beqaa	White pericarp	TaMYC1w
87	Triticum uratu	PI428272	G3155	Lebanon,EI Beqaa	White pericarp	TaMYC1w
88	Triticum uratu	PI428273	G3156	Lebanon,EI Beqaa	White pericarp	TaMYC1w
89	Triticum uratu	PI428274	G3157	Lebanon,EI Beqaa	White pericarp	TaMYC1w
90	Triticum uratu	PI428275	G3158	Lebanon,EI Beqaa	White pericarp	TaMYC1w
91	Triticum uratu	PI428277	G3160	Lebanon,EI Beqaa	White pericarp	TaMYC1w
92	Triticum uratu	PI428278	G3161	Lebanon,EI Beqaa	White pericarp	TaMYC1w
93	Triticum uratu	PI428280	G3163	Lebanon,EI Beqaa	White pericarp	TaMYC1w
94	Triticum uratu	PI428281	G3164	Lebanon,EI Beqaa	White pericarp	TaMYC1w
95	Triticum uratu	PI428282	G3165	Lebanon,EI Beqaa	White pericarp	TaMYC1w
96	Triticum uratu	PI428283	G3166	Lebanon,EI Beqaa	White pericarp	TaMYC1w
97	Triticum uratu	PI428284	G3167	Lebanon,EI Beqaa	White pericarp	TaMYC1w

98	Triticum uratu	PI428285	G3168	Lebanon,EI Beqaa	White pericarp	TaMYC1w
99	Triticum turgidum subsp. durum	CItr14429	57b	Ethiopia, Shewa	Puprle pericarp	TaMYC1p
100	Triticum turgidum subsp. durum	CItr14432	286b	Ethiopia, Shewa	Puprle pericarp	TaMYC1p
101	Triticum turgidum subsp. durum	CItr14435	378b	Ethiopia, Shewa	Puprle pericarp	TaMYC1p
102	Triticum turgidum subsp. durum	CItr14440	665b	Ethiopia, Shewa	Puprle pericarp	TaMYC1p
103	Triticum turgidum subsp. durum	CItr14618	ELS 6304-69	Ethiopia, Shewa	Puprle pericarp	TaMYC1p
104	Triticum turgidum subsp. durum	CItr14629	ELS 6404-75-3	Ethiopia, Shewa	Puprle pericarp	TaMYC1p
105	Triticum turgidum subsp. durum	CItr14708	ELS 6404-98-1	Ethiopia, Harer	Puprle pericarp	TaMYC1p
106	Triticum turgidum subsp. durum	CItr14753	ELS 6404-109	Ethiopia, Gojam	Puprle pericarp	TaMYC1p
107	Triticum turgidum subsp. durum	CItr14754	ELS 6404-110-1	Ethiopia, Shewa	Puprle pericarp	TaMYC1p
108	Triticum turgidum subsp. durum	CItr14755	ELS 6404-110-2	Ethiopia, Shewa	Puprle pericarp	TaMYC1p
109	Triticum turgidum subsp. durum	CItr14758	ELS 6404-111-1	Ethiopia, Gonder	Puprle pericarp	TaMYC1p
110	Triticum turgidum subsp. durum	CItr14765	ELS 6404-114-4	Ethiopia, Gonder	Puprle pericarp	TaMYC1p
111	Triticum turgidum subsp. durum	CItr14768	ELS 6404-115-3	Ethiopia, Gonder	Puprle pericarp	TaMYC1p
112	Triticum turgidum subsp. durum	CItr14770	ELS 6404-115-5	Ethiopia, Gonder	Puprle pericarp	TaMYC1p
113	Triticum turgidum subsp. durum	CItr14774	ELS 6404-116-3	Ethiopia, Gonder	Puprle pericarp	TaMYC1p
114	Triticum turgidum subsp. durum	CItr14786	ELS 6404-118-1	Ethiopia, Gonder	Puprle pericarp	TaMYC1p
115	Triticum turgidum subsp. durum	CItr14813	ELS 6404-126-2	Eritrea	Puprle pericarp	TaMYC1p
116	Triticum turgidum subsp. durum	CItr14814	ELS 6404-126-3	Eritrea	Puprle pericarp	TaMYC1p
117	Triticum turgidum subsp. durum	CItr14825	ELS 6404-130-1	Ethiopia, Tigre	Puprle pericarp	TaMYC1p
118	Triticum turgidum subsp. durum	CItr14827	ELS 6404-130-3	Ethiopia, Tigre	Puprle pericarp	TaMYC1p
119	Triticum turgidum subsp. durum	CItr14850	ELS 6404-139-3	Ethiopia, Shewa	Puprle pericarp	TaMYC1p
120	Triticum turgidum subsp. durum	CItr14859	ELS 6404-140-2	Ethiopia, Shewa	Puprle pericarp	TaMYC1p
121	Triticum turgidum subsp. durum	CItr14862	ELS 6404-140-5	Ethiopia, Shewa	Puprle pericarp	TaMYC1p
122	Triticum turgidum subsp. durum	CItr14899	ELS 6404-158-3	Ethiopia, Kefa	Puprle pericarp	TaMYC1p

123	Triticum turgidum subsp. durum	CItr15168	Schimperi Perier	United States, Minnesota	Puprle pericarp	TaMYC1p
124	Triticum turgidum subsp. durum	CItr17240	MP 3	Ethiopia	Puprle pericarp	TaMYC1p
125	Triticum turgidum subsp. durum	CItr17346	World Seeds 3	United States, California	Puprle pericarp	TaMYC1p
126	Triticum turgidum subsp. durum	CItr17612	Qualset BYDV 3	Ethiopia, Shewa	Puprle pericarp	TaMYC1p
127	Triticum turgidum subsp. durum	CItr17613	Qualset BYDV 4	Ethiopia, Shewa	Puprle pericarp	TaMYC1p
128	Triticum turgidum subsp. durum	CItr17616	Qualset BYDV 7	Ethiopia, Gonder	Puprle pericarp	TaMYC1p
129	Triticum turgidum subsp. durum	CItr17626	Qualset BYDV 17	Ethiopia, Shewa	Puprle pericarp	TaMYC1p
130	Triticum turgidum subsp. durum	CItr17636	Qualset BYDV 27	Ethiopia, Gonder	Puprle pericarp	TaMYC1p
131	Triticum turgidum subsp. durum	CItr17637	Qualset BYDV 28	Ethiopia, Gonder	Puprle pericarp	TaMYC1p
132	Triticum turgidum subsp. durum	CItr17638	Qualset BYDV 29	Ethiopia, Gonder	Puprle pericarp	TaMYC1p
133	Triticum turgidum subsp. durum	PI58792	320	Ethiopia, Shewa	Puprle pericarp	TaMYC1p
134	Triticum turgidum subsp. durum	PI60602	436	Ethiopia, Shewa	Puprle pericarp	TaMYC1p
135	Triticum turgidum subsp. durum	PI94578	20248	Egypt	Puprle pericarp	TaMYC1p
136	Triticum turgidum subsp. durum	PI133183	839	Ethiopia, Kefa	Puprle pericarp	TaMYC1p
137	Triticum turgidum subsp. durum	PI192120	Dabat	Ethiopia, Gonder	Puprle pericarp	TaMYC1p
138	Triticum turgidum subsp. durum	PI192165	Dabat	Ethiopia, Gonder	Puprle pericarp	TaMYC1p
139	Triticum turgidum subsp. durum	PI192594	Tucur	Ethiopia	Puprle pericarp	TaMYC1p
140	Triticum turgidum subsp. durum	PI192620	Moggio	Ethiopia, Shewa	Puprle pericarp	TaMYC1p
141	Triticum turgidum subsp. durum	PI193859	8793	Ethiopia, Shewa	Puprle pericarp	TaMYC1p
142	Triticum turgidum subsp. durum	PI193865	8828	Ethiopia, Shewa	Puprle pericarp	TaMYC1p
143	Triticum turgidum subsp. durum	PI193884	8820	Ethiopia, Shewa	Puprle pericarp	TaMYC1p
144	Triticum turgidum subsp. durum	PI193888	8915	Ethiopia, Shewa	Puprle pericarp	TaMYC1p
145	Triticum turgidum subsp. durum	PI193890	8819	Ethiopia, Shewa	Puprle pericarp	TaMYC1p
146	Triticum turgidum subsp. durum	PI194034	9000	Ethiopia, Shewa	Puprle pericarp	TaMYC1p
147	Triticum turgidum subsp. durum	PI194043	8939	Ethiopia, Shewa	Puprle pericarp	TaMYC1p

148	Triticum turgidum subsp. durum	PI194363	9082	Ethiopia, Kefa	Puprle pericarp	TaMYC1p
149	Triticum turgidum subsp. durum	PI195090	9306	Ethiopia, Gonder	Puprle pericarp	TaMYC1p
150	Triticum turgidum subsp. durum	PI195095	9312	Ethiopia, Gonder	Puprle pericarp	TaMYC1p
151	Triticum turgidum subsp. durum	PI195098	9358	Ethiopia, Gonder	Puprle pericarp	TaMYC1p
152	Triticum turgidum subsp. durum	PI195099	9448	Ethiopia, Gonder	Puprle pericarp	TaMYC1p
153	Triticum turgidum subsp. durum	PI195710	9605	Ethiopia	Puprle pericarp	TaMYC1p
154	Triticum turgidum subsp. durum	PI195725	9592	Ethiopia, Shewa	Puprle pericarp	TaMYC1p
155	Triticum turgidum subsp. durum	PI195726	9594	Ethiopia, Shewa	Puprle pericarp	TaMYC1p
156	Triticum turgidum subsp. durum	PI196081	9652	Ethiopia, Welo	Puprle pericarp	TaMYC1p
157	Triticum turgidum subsp. durum	PI196909	10093	Ethiopia, Shewa	Puprle pericarp	TaMYC1p
158	Triticum turgidum subsp. durum	PI226573	405	Ethiopia, Shewa	Puprle pericarp	TaMYC1p
159	Triticum turgidum subsp. durum	PI234869	15	Ethiopia	Puprle pericarp	TaMYC1p
160	Triticum turgidum subsp. durum	PI244342	Market Sample A	Ethiopia	Puprle pericarp	TaMYC1p
161	Triticum turgidum subsp. durum	PI273974	1842	Ethiopia, Harer	Puprle pericarp	TaMYC1p
162	Triticum turgidum subsp. durum	PI273976	1938	Ethiopia, Shewa	Puprle pericarp	TaMYC1p
163	Triticum turgidum subsp. durum	PI273994	1941	Ethiopia, Shewa	Puprle pericarp	TaMYC1p
164	Triticum turgidum subsp. durum	PI273995	1942	Ethiopia, Shewa	Puprle pericarp	TaMYC1p
165	Triticum turgidum subsp. durum	PI282912	Granos Purpuras	Argentina, Buenos Aires	Puprle pericarp	TaMYC1p
166	Triticum turgidum subsp. durum	PI297839	ELS 6304-6-A	Ethiopia, Shewa	Puprle pericarp	TaMYC1p
167	Triticum turgidum subsp. durum	PI297840	ELS 6304-6-B	Ethiopia, Shewa	Puprle pericarp	TaMYC1p
168	Triticum turgidum subsp. durum	PI297843	ELS 6304-7-B	Ethiopia, Shewa	Puprle pericarp	TaMYC1p
169	Triticum turgidum subsp. durum	PI297857	ELS 6404-9-C	Ethiopia, Bale	Puprle pericarp	TaMYC1p
170	Triticum turgidum subsp. durum	PI298542	ELS 6304-10	Ethiopia, Shewa	Puprle pericarp	TaMYC1p
171	Triticum turgidum subsp. durum	PI298547	ELS 6304-38	Ethiopia, Shewa	Puprle pericarp	TaMYC1p
172	Triticum turgidum subsp. durum	PI298557	ELS 6404-20	Ethiopia, Shewa	Puprle pericarp	TaMYC1p

173	Triticum turgidum subsp. durum	PI298568	ELS 6404-31	Ethiopia, Arusi	Puprle pericarp	TaMYC1p
174	Triticum turgidum subsp. durum	PI298574	ELS-6404-42	Ethiopia, Shewa	Puprle pericarp	TaMYC1p
175	Triticum turgidum subsp. durum	PI298587	ELS 6404-55	Ethiopia, Bale	Puprle pericarp	TaMYC1p
176	Triticum turgidum subsp. durum	PI324937	BD 1588	Ethiopia	Puprle pericarp	TaMYC1p
177	Triticum turgidum subsp. durum	PI331255	R-25	Ethiopia, Kefa	Puprle pericarp	TaMYC1p
178	Triticum turgidum subsp. durum	PI331263	P-496	Ethiopia, Bale	Puprle pericarp	TaMYC1p
179	Triticum turgidum subsp. durum	PI337704	R-67	Ethiopia, Shewa	Puprle pericarp	TaMYC1p
180	Triticum turgidum subsp. durum	PI352394	T-1297	Ethiopia	Puprle pericarp	TaMYC1p
181	Triticum turgidum subsp. durum	PI352395	T-1303	Ethiopia	Puprle pericarp	TaMYC1p
182	Triticum turgidum subsp. durum	PI384076	GAW 4-2	Ethiopia, Gonder	Puprle pericarp	TaMYC1p
183	Triticum turgidum subsp. durum	PI384098	GAW 8-2	Ethiopia, Gonder	Puprle pericarp	TaMYC1p
184	Triticum turgidum subsp. durum	PI384105	GAW 9-2	Ethiopia, Gonder	Puprle pericarp	TaMYC1p
185	Triticum turgidum subsp. durum	PI384110	GAW 10-1	Ethiopia, Gonder	Puprle pericarp	TaMYC1p
186	Triticum turgidum subsp. durum	PI384113	GAW 10-4	Ethiopia, Gonder	Puprle pericarp	TaMYC1p
187	Triticum turgidum subsp. durum	PI384114	GAW 10-5	Ethiopia, Gonder	Puprle pericarp	TaMYC1p
188	Triticum turgidum subsp. durum	PI384116	GAW 11-1	Ethiopia, Gonder	Puprle pericarp	TaMYC1p
189	Triticum turgidum subsp. durum	PI384117	GAW 11-2	Ethiopia, Gonder	Puprle pericarp	TaMYC1p
190	Triticum turgidum subsp. durum	PI384120	GAW 11-5	Ethiopia, Gonder	Puprle pericarp	TaMYC1p
191	Triticum turgidum subsp. durum	PI384125	GAW 11-10	Ethiopia, Gonder	Puprle pericarp	TaMYC1p
192	Triticum turgidum subsp. durum	PI384127	GAW 11-12	Ethiopia, Gonder	Puprle pericarp	TaMYC1p
193	Triticum turgidum subsp. durum	PI384129	GAW 12-2	Ethiopia, Gonder	Puprle pericarp	TaMYC1p
194	Triticum turgidum subsp. durum	PI384132	GAW 14-1	Ethiopia, Gonder	Puprle pericarp	TaMYC1p
195	Triticum turgidum subsp. durum	PI384133	GAW 14-2	Ethiopia, Gonder	Puprle pericarp	TaMYC1p
196	Triticum turgidum subsp. durum	PI384138	GAW 15-2	Ethiopia, Gonder	Puprle pericarp	TaMYC1p
197	Triticum turgidum subsp. durum	PI384140	GAW 15-4	Ethiopia, Gonder	Puprle pericarp	TaMYC1p

198	Triticum turgidum subsp. durum	PI384141	GAW 15-5	Ethiopia, Gonder	Puprle pericarp	TaMYC1p
199	Triticum turgidum subsp. durum	PI384144	GAW 16-2	Ethiopia, Gonder	Puprle pericarp	TaMYC1p
200	Triticum turgidum subsp. durum	PI384147	GAW 18-2	Ethiopia, Gonder	Puprle pericarp	TaMYC1p
201	Triticum turgidum subsp. durum	PI384150	GAW 18-5	Ethiopia, Gonder	Puprle pericarp	TaMYC1p
202	Triticum turgidum subsp. durum	PI384153	GAW 19-1	Ethiopia, Gonder	Puprle pericarp	TaMYC1p
203	Triticum turgidum subsp. durum	PI384154	GAW 19-2	Ethiopia, Gonder	Puprle pericarp	TaMYC1p
204	Triticum turgidum subsp. durum	PI384160	GAW 20-4	Ethiopia, Gonder	Puprle pericarp	TaMYC1p
205	Triticum turgidum subsp. durum	PI384162	GAW 20-6	Ethiopia, Gonder	Puprle pericarp	TaMYC1p
206	Triticum turgidum subsp. durum	PI384167	GAW 21-5	Ethiopia, Gonder	Puprle pericarp	TaMYC1p
207	Triticum turgidum subsp. durum	PI384196	GAW 27-1	Ethiopia, Tigre	Puprle pericarp	TaMYC1p
208	Triticum turgidum subsp. durum	PI384199	GAW 27-5	Ethiopia, Tigre	Puprle pericarp	TaMYC1p
209	Triticum turgidum subsp. durum	PI384248	GAW 38-1	Ethiopia, Shewa	Puprle pericarp	TaMYC1p
210	Triticum turgidum subsp. durum	PI384249	GAW 38-2	Ethiopia, Shewa	Puprle pericarp	TaMYC1p
211	Triticum turgidum subsp. durum	PI384272	GAW 43-2	Ethiopia, Shewa	Puprle pericarp	TaMYC1p
212	Triticum turgidum subsp. durum	PI384276	GAW 44-2	Ethiopia, Shewa	Puprle pericarp	TaMYC1p
213	Triticum turgidum subsp. durum	PI384285	GAW 46-9	Ethiopia, Welega	Puprle pericarp	TaMYC1p
214	Triticum turgidum subsp. durum	PI384286	GAW 46-10	Ethiopia, Welega	Puprle pericarp	TaMYC1p
215	Triticum turgidum subsp. durum	PI384295	GAW 50-2	Ethiopia, Shewa	Puprle pericarp	TaMYC1p
216	Triticum turgidum subsp. durum	PI384326	GAW 45-13	Ethiopia, Shewa	Puprle pericarp	TaMYC1p
217	Triticum turgidum subsp. durum	PI387251	IAR/W/1-1	Ethiopia	Puprle pericarp	TaMYC1p
218	Triticum turgidum subsp. durum	PI387259	IAR/W/3-4	Ethiopia	Puprle pericarp	TaMYC1p
219	Triticum turgidum subsp. durum	PI387261	IAR/W/4-1	Ethiopia	Puprle pericarp	TaMYC1p
220	Triticum turgidum subsp. durum	PI387264	IAR/W/5-1	Ethiopia	Puprle pericarp	TaMYC1p
221	Triticum turgidum subsp. durum	PI387278	IAR/W/10-1	Ethiopia	Puprle pericarp	TaMYC1p
222	Triticum turgidum subsp. durum	PI387281	IAR/W/11-1	Ethiopia	Puprle pericarp	TaMYC1p

223	Triticum turgidum subsp. durum	PI387297	IAR/W/18-1	Ethiopia	Puprle pericarp	TaMYC1p
224	Triticum turgidum subsp. durum	PI387306	IAR/W/22-2	Ethiopia	Puprle pericarp	TaMYC1p
225	Triticum turgidum subsp. durum	PI387309	IAR/W/24-1	Ethiopia	Puprle pericarp	TaMYC1p
226	Triticum turgidum subsp. durum	PI387312	IAR/W/25-2	Ethiopia	Puprle pericarp	TaMYC1p
227	Triticum turgidum subsp. durum	PI387321	IAR/W/27-2	Ethiopia	Puprle pericarp	TaMYC1p
228	Triticum turgidum subsp. durum	PI387330	IAR/W/31-1	Ethiopia	Puprle pericarp	TaMYC1p
229	Triticum turgidum subsp. durum	PI387370	IAR/W/44-2	Ethiopia	Puprle pericarp	TaMYC1p
230	Triticum turgidum subsp. durum	PI387376	IAR/W/47-1	Ethiopia	Puprle pericarp	TaMYC1p
231	Triticum turgidum subsp. durum	PI387386	IAR/W/50-1	Ethiopia	Puprle pericarp	TaMYC1p
232	Triticum turgidum subsp. durum	PI387401	IAR/W/55-3	Ethiopia	Puprle pericarp	TaMYC1p
233	Triticum turgidum subsp. durum	PI387407	IAR/W/57-2	Ethiopia	Puprle pericarp	TaMYC1p
234	Triticum turgidum subsp. durum	PI387443	IAR/W/70-3	Ethiopia	Puprle pericarp	TaMYC1p
235	Triticum turgidum subsp. durum	PI387445	IAR/W/71	Ethiopia	Puprle pericarp	TaMYC1p
236	Triticum turgidum subsp. durum	PI387468	IAR/W/80-2	Ethiopia	Puprle pericarp	TaMYC1p
237	Triticum turgidum subsp. durum	PI387470	IAR/W/81-1	Ethiopia	Puprle pericarp	TaMYC1p
238	Triticum turgidum subsp. durum	PI387478	IAR/W/84-2	Ethiopia	Puprle pericarp	TaMYC1p
239	Triticum turgidum subsp. durum	PI387513	IAR/W/96-3	Ethiopia	Puprle pericarp	TaMYC1p
240	Triticum turgidum subsp. durum	PI387570	IAR/W/120	Ethiopia	Puprle pericarp	TaMYC1p
241	Triticum turgidum subsp. durum	PI387608	IAR/W/134-1	Ethiopia	Puprle pericarp	TaMYC1p
242	Triticum turgidum subsp. durum	PI387625	IAR/W/140-2	Ethiopia	Puprle pericarp	TaMYC1p
243	Triticum turgidum subsp. durum	PI387658	IAR/W/153-4	Ethiopia	Puprle pericarp	TaMYC1p
244	Triticum turgidum subsp. durum	PI387707	IAR/W/174-3	Ethiopia	Puprle pericarp	TaMYC1p
245	Triticum turgidum subsp. durum	PI387719	IAR/W/180-1	Ethiopia	Puprle pericarp	TaMYC1p
246	Triticum turgidum subsp. durum	PI470785	MG 7852	Ethiopia, Shewa	Puprle pericarp	TaMYC1p
247	Triticum turgidum subsp. durum	PI470786	MG 7853	Ethiopia, Shewa	Puprle pericarp	TaMYC1p

248	Triticum turgidum subsp. durum	PI470800	MG 7878	Ethiopia	Puprle pericarp	TaMYC1p
249	Triticum turgidum subsp. durum	PI470808	MG 15879	Ethiopia, Shewa	Puprle pericarp	TaMYC1p
250	Triticum turgidum subsp. durum	PI479913	MG 31004	Ethiopia, Shewa	Puprle pericarp	TaMYC1p
251	Triticum turgidum subsp. durum	PI479920	MG 31011	Ethiopia, Shewa	Puprle pericarp	TaMYC1p
252	Triticum turgidum subsp. durum	PI479924	MG 31016	Ethiopia, Shewa	Puprle pericarp	TaMYC1p
253	Triticum turgidum subsp. durum	PI479925	MG 31017	Ethiopia, Shewa	Puprle pericarp	TaMYC1p
254	Triticum turgidum subsp. durum	PI479928	MG 31020	Ethiopia, Shewa	Puprle pericarp	TaMYC1p
255	Triticum turgidum subsp. durum	PI479939	MG 31031	Ethiopia, Shewa	Puprle pericarp	TaMYC1p
256	Triticum turgidum subsp. durum	PI479951	MG 31044	Ethiopia, Shewa	Puprle pericarp	TaMYC1p
257	Triticum turgidum subsp. durum	PI479975	MG 31070	Ethiopia, Shewa	Puprle pericarp	TaMYC1p
258	Triticum turgidum subsp. durum	PI479980	MG 31078	Ethiopia, Shewa	Puprle pericarp	TaMYC1p
259	Triticum turgidum subsp. durum	PI479982	MG 31080	Ethiopia, Shewa	Puprle pericarp	TaMYC1p
260	Triticum turgidum subsp. durum	PI479985	MG 31083	Ethiopia, Shewa	Puprle pericarp	TaMYC1p
261	Triticum turgidum subsp. durum	PI479986	MG 31084	Ethiopia, Shewa	Puprle pericarp	TaMYC1p
262	Triticum turgidum subsp. durum	PI479989	MG 31087	Ethiopia, Shewa	Puprle pericarp	TaMYC1p
263	Triticum turgidum subsp. durum	PI479991	MG 31090	Ethiopia, Shewa	Puprle pericarp	TaMYC1p
264	Triticum turgidum subsp. durum	PI479993	MG 31092	Ethiopia, Shewa	Puprle pericarp	TaMYC1p
265	Triticum turgidum subsp. durum	PI479994	MG 31093	Ethiopia, Shewa	Puprle pericarp	TaMYC1p
266	Triticum turgidum subsp. durum	PI479996	MG 31095	Ethiopia, Shewa	Puprle pericarp	TaMYC1p
267	Triticum turgidum subsp. durum	PI479998	MG 31097	Ethiopia, Shewa	Puprle pericarp	TaMYC1p
268	Triticum turgidum subsp. durum	PI480001	MG 31100	Ethiopia, Shewa	Puprle pericarp	TaMYC1p
269	Triticum turgidum subsp. durum	PI480002	MG 31101	Ethiopia, Shewa	Puprle pericarp	TaMYC1p
270	Triticum turgidum subsp. durum	PI480006	MG 31105	Ethiopia, Shewa	Puprle pericarp	TaMYC1p
271	Triticum turgidum subsp. durum	PI480017	MG 31117	Ethiopia, Shewa	Puprle pericarp	TaMYC1p
272	Triticum turgidum subsp. durum	PI480018	MG 31118	Ethiopia, Shewa	Puprle pericarp	TaMYC1p

273	Triticum turgidum subsp. durum	PI480026	MG 31129	Ethiopia, Shewa	Puprle pericarp	TaMYC1p
274	Triticum turgidum subsp. durum	PI480036	MG 31151	Ethiopia, Shewa	Puprle pericarp	TaMYC1p
275	Triticum turgidum subsp. durum	PI480038	MG 31153	Ethiopia, Shewa	Puprle pericarp	TaMYC1p
276	Triticum turgidum subsp. durum	PI480046	MG 31162	Ethiopia, Shewa	Puprle pericarp	TaMYC1p
277	Triticum turgidum subsp. durum	PI480047	MG 31163	Ethiopia, Shewa	Puprle pericarp	TaMYC1p
278	Triticum turgidum subsp. durum	PI480048	MG 31164	Ethiopia, Shewa	Puprle pericarp	TaMYC1p
279	Triticum turgidum subsp. durum	PI480050	MG 31166	Ethiopia, Shewa	Puprle pericarp	TaMYC1p
280	Triticum turgidum subsp. durum	PI480051	MG 31167	Ethiopia, Shewa	Puprle pericarp	TaMYC1p
281	Triticum turgidum subsp. durum	PI480052	MG 31168	Ethiopia, Shewa	Puprle pericarp	TaMYC1p
282	Triticum turgidum subsp. durum	PI480053	MG 31169	Ethiopia, Shewa	Puprle pericarp	TaMYC1p
283	Triticum turgidum subsp. durum	PI480054	MG 31170	Ethiopia, Shewa	Puprle pericarp	TaMYC1p
284	Triticum turgidum subsp. durum	PI480056	MG 31172	Ethiopia, Shewa	Puprle pericarp	TaMYC1p
285	Triticum turgidum subsp. durum	PI480057	MG 31173	Ethiopia, Shewa	Puprle pericarp	TaMYC1p
286	Triticum turgidum subsp. durum	PI480059	MG 31176	Ethiopia, Shewa	Puprle pericarp	TaMYC1p
287	Triticum turgidum subsp. durum	PI480061	MG 31178	Ethiopia, Shewa	Puprle pericarp	TaMYC1p
288	Triticum turgidum subsp. durum	PI480062	MG 31179	Ethiopia, Shewa	Puprle pericarp	TaMYC1p
289	Triticum turgidum subsp. durum	PI480064	MG 31181	Ethiopia, Shewa	Puprle pericarp	TaMYC1p
290	Triticum turgidum subsp. durum	PI480066	MG 31183	Ethiopia, Shewa	Puprle pericarp	TaMYC1p
291	Triticum turgidum subsp. durum	PI480070	MG 31188	Ethiopia, Shewa	Puprle pericarp	TaMYC1p
292	Triticum turgidum subsp. durum	PI480071	MG 31189	Ethiopia, Shewa	Puprle pericarp	TaMYC1p
293	Triticum turgidum subsp. durum	PI480073	MG 31191	Ethiopia, Shewa	Puprle pericarp	TaMYC1p
294	Triticum turgidum subsp. durum	PI480074	MG 31192	Ethiopia, Shewa	Puprle pericarp	TaMYC1p
295	Triticum turgidum subsp. durum	PI480075	MG 31193	Ethiopia, Shewa	Puprle pericarp	TaMYC1p
296	Triticum turgidum subsp. durum	PI480076	MG 31194	Ethiopia, Shewa	Puprle pericarp	TaMYC1p
297	Triticum turgidum subsp. durum	PI480079	MG 31197	Ethiopia, Shewa	Puprle pericarp	TaMYC1p

298	Triticum turgidum subsp. durum	PI480082	MG 31201	Ethiopia, Shewa	Puprle pericarp	TaMYC1p
299	Triticum turgidum subsp. durum	PI480084	MG 31203	Ethiopia, Shewa	Puprle pericarp	TaMYC1p
300	Triticum turgidum subsp. durum	PI480104	MG 31224	Ethiopia, Shewa	Puprle pericarp	TaMYC1p
301	Triticum turgidum subsp. durum	PI480106	MG 31226	Ethiopia, Shewa	Puprle pericarp	TaMYC1p
302	Triticum turgidum subsp. durum	PI480108	MG 31229	Ethiopia, Shewa	Puprle pericarp	TaMYC1p
303	Triticum turgidum subsp. durum	PI480110	MG 31231	Ethiopia, Shewa	Puprle pericarp	TaMYC1p
304	Triticum turgidum subsp. durum	PI480111	MG 31232	Ethiopia, Shewa	Puprle pericarp	TaMYC1p
305	Triticum turgidum subsp. durum	PI480115	MG 31237	Ethiopia, Shewa	Puprle pericarp	TaMYC1p
306	Triticum turgidum subsp. durum	PI480116	MG 31238	Ethiopia, Shewa	Puprle pericarp	TaMYC1p
307	Triticum turgidum subsp. durum	PI480119	MG 31241	Ethiopia, Shewa	Puprle pericarp	TaMYC1p
308	Triticum turgidum subsp. durum	PI480131	MG 31253	Ethiopia, Shewa	Puprle pericarp	TaMYC1p
309	Triticum turgidum subsp. durum	PI480148	MG 31274	Ethiopia, Shewa	Puprle pericarp	TaMYC1p
310	Triticum turgidum subsp. durum	PI480149	MG 31275	Ethiopia, Shewa	Puprle pericarp	TaMYC1p
311	Triticum turgidum subsp. durum	PI480150	MG 31279	Ethiopia, Shewa	Puprle pericarp	TaMYC1p
312	Triticum turgidum subsp. durum	PI480181	MG 31320	Ethiopia	Puprle pericarp	TaMYC1p
313	Triticum turgidum subsp. durum	PI480191	MG 31358	Ethiopia	Puprle pericarp	TaMYC1p
314	Triticum turgidum subsp. durum	PI480192	MG 31360	Ethiopia	Puprle pericarp	TaMYC1p
315	Triticum turgidum subsp. durum	PI480243	MG 31455	Ethiopia	Puprle pericarp	TaMYC1p
316	Triticum turgidum subsp. durum	PI480249	MG 31467	Ethiopia	Puprle pericarp	TaMYC1p
317	Triticum turgidum subsp. durum	PI480259	MG 31478	Ethiopia	Puprle pericarp	TaMYC1p
318	Triticum turgidum subsp. durum	PI480260	MG 31479	Ethiopia	Puprle pericarp	TaMYC1p
319	Triticum turgidum subsp. durum	PI480288	MG 31510	Ethiopia	Puprle pericarp	TaMYC1p
320	Triticum turgidum subsp. durum	PI480295	MG 31517	Ethiopia	Puprle pericarp	TaMYC1p
321	Triticum turgidum subsp. durum	PI480296	MG 31518	Ethiopia	Puprle pericarp	TaMYC1p
322	Triticum turgidum subsp. durum	PI480306	MG 31535	Ethiopia, Shewa	Puprle pericarp	TaMYC1p

323	Triticum turgidum subsp. durum	PI480319	MG 31549	Ethiopia, Shewa	Puprle pericarp	TaMYC1p
324	Triticum turgidum subsp. durum	PI480322	MG 31552	Ethiopia, Shewa	Puprle pericarp	TaMYC1p
325	Triticum turgidum subsp. durum	PI480326	MG 31556	Ethiopia, Shewa	Puprle pericarp	TaMYC1p
326	Triticum turgidum subsp. durum	PI480406	MG 31659	Ethiopia, Shewa	Puprle pericarp	TaMYC1p
327	Triticum turgidum subsp. durum	PI480410	MG 31663	Ethiopia, Shewa	Puprle pericarp	TaMYC1p
328	Triticum turgidum subsp. durum	PI480445	MG 31699	Ethiopia, Shewa	Puprle pericarp	TaMYC1p
329	Triticum turgidum subsp. durum	PI480447	MG 31701	Ethiopia, Shewa	Puprle pericarp	TaMYC1p
330	Triticum turgidum subsp. durum	PI519963	Condore 839	Ethiopia	Puprle pericarp	TaMYC1p
331	Triticum turgidum subsp. durum	PI534278	MG 07719	Ethiopia, Arusi	Puprle pericarp	TaMYC1p
332	Triticum turgidum subsp. durum	PI534280	MG 07724	Ethiopia, Arusi	Puprle pericarp	TaMYC1p
333	Triticum turgidum subsp. durum	PI534287	MG 07734	Ethiopia, Arusi	Puprle pericarp	TaMYC1p
334	Triticum turgidum subsp. durum	PI534329	Sindi	Ethiopia, Tigre	Puprle pericarp	TaMYC1p
335	Triticum turgidum subsp. durum	PI24324	Rubion	Spain, Salamanca	White pericarp	TaMYC1w
336	Triticum turgidum subsp. durum	CItr13165	Langdon		White pericarp	TaMYC1w
337	Triticum turgidum subsp. durum	AS295			White pericarp	TaMYC1w
338	Triticum turgidum subsp. durum	AS2263		America	White pericarp	TaMYC1w
339	Triticum turgidum subsp. durum	AS2264		Albania	White pericarp	TaMYC1w
340	Triticum turgidum subsp. durum	AS2234		China, Xinjiang	White pericarp	TaMYC1w
341	Triticum turgidum subsp. durum	AS2246		China，Gansu	White pericarp	TaMYC1w
342	Triticum turgidum subsp. durum	AS2247		China，Gansu	White pericarp	TaMYC1w
343	Triticum turgidum subsp. durum	AS2248		China，Gansu	White pericarp	TaMYC1w
344	Triticum turgidum subsp. durum	AS2260		Kenya	White pericarp	TaMYC1w
345	Triticum turgidum subsp. durum	AS2261		Mexico	White pericarp	TaMYC1w
346	Triticum turgidum subsp. durum	AS2264		Albania	White pericarp	TaMYC1w

347	Triticum turgidum subsp. durum	AS2280		China, Xinjiang	White pericarp	TaMYC1w
348	Triticum turgidum subsp. durum		obeirodegranESC		White pericarp	TaMYC1w
349	Triticum turgidum subsp. durum		FLAMINGO		White pericarp	TaMYC1w
350	Triticum turgidum subsp. durum		PETRE		White pericarp	TaMYC1w
351	Triticum turgidum subsp. durum		CH-3002-7P-3P-11A		White pericarp	TaMYC1w
352	Triticum turgidum subsp. durum		MACEDONIA		White pericarp	TaMYC1w
353	Triticum turgidum subsp. durum		kronos		White pericarp	TaMYC1w
354	Triticum turgidum subsp. durum		kafa		White pericarp	TaMYC1w
355	Triticum turgidum subsp. polonicum	PI384344	GAW 45-8	Ethiopia, Shewa	Puprle pericarp	TaMYC1p
356	Triticum turgidum subsp. polonicum	PI384345	GAW 47-2	Ethiopia, Shewa	Puprle pericarp	TaMYC1p
357	Triticum turgidum subsp. polonicum	PI56262			White pericarp	TaMYC1w
358	Triticum turgidum subsp. polonicum	PI14139			White pericarp	TaMYC1w
359	Triticum turgidum subsp. polonicum	PI14140			White pericarp	TaMYC1w
360	Triticum turgidum subsp. turgidum	CItr14863	ELS 6404-141-1	Ethiopia, Shewa	Puprle pericarp	TaMYC1p
361	Triticum turgidum subsp. turgidum	PI213571	Halcon	Argentina	Puprle pericarp	TaMYC1p
362	Triticum turgidum subsp. turgidum	PI297860	ELS 6304-8-F	Ethiopia, Shewa	Puprle pericarp	TaMYC1p
363	Triticum turgidum subsp. turgidum	PI331258	R-74	Ethiopia, Shewa	Puprle pericarp	TaMYC1p
364	Triticum turgidum subsp. turgidum	PI387336	IAR/W/33-1	Ethiopia	Puprle pericarp	TaMYC1p
365	Triticum turgidum subsp. turgidum	PI479946	MG 31038	Ethiopia, Shewa	Puprle pericarp	TaMYC1p
366	Triticum turgidum subsp. turgidum	PI480041	MG 31156	Ethiopia, Shewa	Puprle pericarp	TaMYC1p
367	Triticum turgidum subsp. turgidum	PI480072	MG 31190	Ethiopia, Shewa	Puprle pericarp	TaMYC1p
368	Triticum turgidum subsp. turgidum	PI520175	Burgas 2	Bulgaria	Puprle pericarp	TaMYC1p
369	Triticum turgidum subsp. turgidum	PI559977	1939	Ethiopia, Shewa	Puprle pericarp	TaMYC1p
370	Triticum turgidum subsp. turgidum	PI352549	Campania 21	Italy	White pericarp	TaMYC1w
371	Triticum turgidum subsp. turgidum	AS2238			White pericarp	TaMYC1w

372	Triticum turgidum subsp. turgidum	AS2239		China, Sichuan	White pericarp	TaMYC1w
373	Triticum turgidum subsp. turgidum	AS2240		China, Sichuan	White pericarp	TaMYC1w
374	Triticum turgidum subsp. turgidum	AS2255			White pericarp	TaMYC1w
375	Triticum turgidum subsp. turgidum	AS313			White pericarp	TaMYC1w
376	Triticum turgidum subsp. turgidum	AS2295		China, Sichuan	White pericarp	TaMYC1w
377	Triticum turgidum subsp. turgidum	AS2296		China, Sichuan	White pericarp	TaMYC1w
378	Triticum turgidum subsp. turgidum	AS2326		China, Gansu	White pericarp	TaMYC1w
379	Triticum turgidum subsp. turgidum	AS2378		China, Shanxi	White pericarp	TaMYC1w
380	Triticum turgidum subsp. turgidum	AS2380		China, Shanxi	White pericarp	TaMYC1w
381	Triticum turgidum subsp. turgidum	AS2382		China, Shanxi	White pericarp	TaMYC1w
382	Triticum turgidum subsp. turgidum	AS2351		China, Henan	White pericarp	TaMYC1w
383	Triticum turgidum subsp. turgidum	AS2238			White pericarp	TaMYC1w
384	Triticum turgidum subsp. turgidum	AS2240		China, Sichuan	White pericarp	TaMYC1w
385	Triticum turgidum subsp. turgidum	AS2249		China, Sichuan	White pericarp	TaMYC1w
386	Triticum turgidum subsp. turgidum	AS2285		China, Sichuan	White pericarp	TaMYC1w
387	Triticum aestivum subsp. aestivum	CItr14952	CI 14952	United States, North Dakota	Puprle pericarp	TaMYC1p
388	Triticum aestivum subsp. aestivum	PI316440		New Zealand, South Island	Puprle pericarp	TaMYC1p
389	Triticum aestivum subsp. aestivum	PI316906	1159.288.0.P1.1.X.1.2	Australia, New South Wales	Puprle pericarp	TaMYC1p
390	Triticum aestivum subsp. aestivum	PI316907	1159.288.18B.1.2.1.2.1<br>3	Australia, New South Wales	Puprle pericarp	TaMYC1p
391	Triticum aestivum subsp. aestivum	PI534281	MG 07726	Ethiopia, Arusi	Puprle pericarp	TaMYC1p
392	Triticum aestivum subsp. aestivum	PI534284	Tucursindi	Ethiopia, Arusi	Puprle pericarp	TaMYC1p
393	Triticum aestivum subsp. aestivum	PI534290	MG 07739	Ethiopia, Arusi	Puprle pericarp	TaMYC1p
394	Triticum aestivum subsp. aestivum	PI534291	MG 07741	Ethiopia, Arusi	Puprle pericarp	TaMYC1p
395	Triticum aestivum subsp. aestivum	PI534296	MG 07756	Ethiopia, Shewa	Puprle pericarp	TaMYC1p

396	Triticum aestivum subsp. aestivum	PI534297	MG 07759	Ethiopia, Shewa	Puprle pericarp	TaMYC1p
397	Triticum aestivum subsp. aestivum	PI534299	MG 07761	Ethiopia, Shewa	Puprle pericarp	TaMYC1p
398	Triticum aestivum subsp. aestivum	PI534302	MG 07766	Ethiopia, Shewa	Puprle pericarp	TaMYC1p
399	Triticum aestivum subsp. aestivum	PI534316	Sindi	Ethiopia, Tigre	Puprle pericarp	TaMYC1p
400	Triticum aestivum subsp. aestivum	PI542453	H86-701	New Zealand	Puprle pericarp	TaMYC1p
401	Triticum aestivum subsp. aestivum		Abo	China, Qinghai	White pericarp	TaMYC1w
402	Triticum aestivum subsp. aestivum		Chaoxuan5	China, Qinghai	White pericarp	TaMYC1w
403	Triticum aestivum subsp. aestivum		Dongchun1	China, Qinghai	White pericarp	TaMYC1w
404	Triticum aestivum subsp. aestivum		Ganchun20	China, Qinghai	White pericarp	TaMYC1w
405	Triticum aestivum subsp. aestivum		Hanhai304	China, Qinghai	White pericarp	TaMYC1w
406	Triticum aestivum subsp. aestivum		Lantian3	China, Qinghai	White pericarp	TaMYC1w
407	Triticum aestivum subsp. aestivum		Ningchun26	China, Qinghai	White pericarp	TaMYC1w
408	Triticum aestivum subsp. aestivum		Shanhan901	China, Qinghai	White pericarp	TaMYC1w
409	Triticum aestivum subsp. aestivum		Tongmai1	China, Qinghai	White pericarp	TaMYC1w
410	Triticum aestivum subsp. aestivum		Xiangnong3	China, Qinghai	White pericarp	TaMYC1w
411	Triticum aestivum subsp. aestivum		Xinze9	China, Qinghai	White pericarp	TaMYC1w
412	Triticum aestivum subsp. aestivum		Yuanzhuo3	China, Qinghai	White pericarp	TaMYC1w
413	Triticum aestivum subsp. aestivum		Zhangchun811	China, Qinghai	White pericarp	TaMYC1w
414	Triticum aestivum subsp. aestivum		Mobo	China, Qinghai	White pericarp	TaMYC1w
415	Triticum aestivum subsp. aestivum		Huzhuhong	China, Qinghai	White pericarp	TaMYC1w
416	Triticum aestivum subsp. aestivum		Lemai5	China, Qinghai	White pericarp	TaMYC1w
417	Triticum aestivum subsp. aestivum		Lemai6	China, Qinghai	White pericarp	TaMYC1w
418	Triticum aestivum subsp. aestivum		Moyin1	China, Qinghai	White pericarp	TaMYC1w
419	Triticum aestivum subsp. aestivum		Moyin2	China, Qinghai	White pericarp	TaMYC1w
420	Triticum aestivum subsp. aestivum		Qingnong469	China, Qinghai	White pericarp	TaMYC1w

421	Triticum aestivum subsp. aestivum		Qingnong524	China, Qinghai	White pericarp	TaMYC1w
422	Triticum aestivum subsp. aestivum		Minghe588	China, Qinghai	White pericarp	TaMYC1w
423	Triticum aestivum subsp. aestivum		Minghe665	China, Qinghai	White pericarp	TaMYC1w
424	Triticum aestivum subsp. aestivum		Minghe853	China, Qinghai	White pericarp	TaMYC1w
425	Triticum aestivum subsp. aestivum		Chaichun018	China, Qinghai	White pericarp	TaMYC1w
426	Triticum aestivum subsp. aestivum		Chaichun044	China, Qinghai	White pericarp	TaMYC1w
427	Triticum aestivum subsp. aestivum		Chaichun236	China, Qinghai	White pericarp	TaMYC1w
428	Triticum aestivum subsp. aestivum		Chaichun901	China, Qinghai	White pericarp	TaMYC1w
429	Triticum aestivum subsp. aestivum		Humai11	China, Qinghai	White pericarp	TaMYC1w
430	Triticum aestivum subsp. aestivum		Humai12	China, Qinghai	White pericarp	TaMYC1w
431	Triticum aestivum subsp. aestivum		Humai13	China, Qinghai	White pericarp	TaMYC1w
432	Triticum aestivum subsp. aestivum		Humai14	China, Qinghai	White pericarp	TaMYC1w
433	Triticum aestivum subsp. aestivum		Humai15	China, Qinghai	White pericarp	TaMYC1w
434	Triticum aestivum subsp. aestivum		Qingchun37	China, Qinghai	White pericarp	TaMYC1w
435	Triticum aestivum subsp. aestivum		Qingchun38	China, Qinghai	White pericarp	TaMYC1w
436	Triticum aestivum subsp. aestivum		Qingchun39	China, Qinghai	White pericarp	TaMYC1w
437	Triticum aestivum subsp. aestivum		Qingchun144	China, Qinghai	White pericarp	TaMYC1w
438	Triticum aestivum subsp. aestivum		Qingchun254	China, Qinghai	White pericarp	TaMYC1w
439	Triticum aestivum subsp. aestivum		Qingchun415	China, Qinghai	White pericarp	TaMYC1w
440	Triticum aestivum subsp. aestivum		Qingchun533	China, Qinghai	White pericarp	TaMYC1w
441	Triticum aestivum subsp. aestivum		Qingchun570	China, Qinghai	White pericarp	TaMYC1w
442	Triticum aestivum subsp. aestivum		Qingchun587	China, Qinghai	White pericarp	TaMYC1w
443	Triticum aestivum subsp. aestivum		Qingchun891	China, Qinghai	White pericarp	TaMYC1w
444	Triticum aestivum subsp. aestivum		Qingchun952	China, Qinghai	White pericarp	TaMYC1w
445	Triticum aestivum subsp. aestivum		Gaoyuanv028	China, Qinghai	White pericarp	TaMYC1w

446	Triticum aestivum subsp. aestivum		Gaoyuan142	China, Qinghai	White pericarp	TaMYC1w
447	Triticum aestivum subsp. aestivum		Gaoyuan158	China, Qinghai	White pericarp	TaMYC1w
448	Triticum aestivum subsp. aestivum		Gaoyuan175	China, Qinghai	White pericarp	TaMYC1w
449	Triticum aestivum subsp. aestivum		Gaoyuan182	China, Qinghai	White pericarp	TaMYC1w
450	Triticum aestivum subsp. aestivum		Gaoyuan205	China, Qinghai	White pericarp	TaMYC1w
451	Triticum aestivum subsp. aestivum		Gaoyuan314	China, Qinghai	White pericarp	TaMYC1w
452	Triticum aestivum subsp. aestivum		Gaoyuan338	China, Qinghai	White pericarp	TaMYC1w
453	Triticum aestivum subsp. aestivum		Gaoyuan356	China, Qinghai	White pericarp	TaMYC1w
454	Triticum aestivum subsp. aestivum		Gaoyuan363	China, Qinghai	White pericarp	TaMYC1w
455	Triticum aestivum subsp. aestivum		Gaoyuan412	China, Qinghai	White pericarp	TaMYC1w
456	Triticum aestivum subsp. aestivum		Gaoyuan437	China, Qinghai	White pericarp	TaMYC1w
457	Triticum aestivum subsp. aestivum		Gaoyuan448	China, Qinghai	White pericarp	TaMYC1w
458	Triticum aestivum subsp. aestivum		Gaoyuan465	China, Qinghai	White pericarp	TaMYC1w
459	Triticum aestivum subsp. aestivum		Gaoyuan466	China, Qinghai	White pericarp	TaMYC1w
460	Triticum aestivum subsp. aestivum		Gaoyuan506	China, Qinghai	White pericarp	TaMYC1w
461	Triticum aestivum subsp. aestivum		Gaoyuan584	China, Qinghai	White pericarp	TaMYC1w
462	Triticum aestivum subsp. aestivum		Gaoyuan602	China, Qinghai	White pericarp	TaMYC1w
463	Triticum aestivum subsp. aestivum		Gaoyuan671	China, Qinghai	White pericarp	TaMYC1w
464	Triticum aestivum subsp. aestivum		Gaoyuan913	China, Qinghai	White pericarp	TaMYC1w
465	Triticum aestivum subsp. aestivum		Gaoyuan932	China, Qinghai	White pericarp	TaMYC1w
466	Triticum aestivum subsp. aestivum		Qingmai1	China, Qinghai	White pericarp	TaMYC1w
467	Triticum aestivum subsp. aestivum	PI542500	R842794	United States,Oregon	White pericarp	TaMYC1w
468	Triticum aestivum subsp. aestivum	PI542501	R842776	United States,Oregon	White pericarp	TaMYC1w
469	Triticum aestivum subsp. aestivum	CItr11731	Kansas No. 343273	United States, Kansas	White pericarp	TaMYC1w
470	Triticum aestivum subsp. aestivum	CItr13537	TX 240-51-A2	United States, Texas	White pericarp	TaMYC1w

471	Triticum aestivum subsp. aestivum	CItr14811	ELS 6404-125	Eritrea, Akele Guzai	White pericarp	TaMYC1w
472	Triticum aestivum subsp. aestivum	CItr17685	Parker 76	United States, Kansas	White pericarp	TaMYC1w
473	Triticum aestivum subsp. aestivum	PI24324	Rubion	Spain, Salamanca	White pericarp	TaMYC1w
474	Triticum aestivum subsp. aestivum	PI57163	CItr 7110	Georgia	White pericarp	TaMYC1w
475	Triticum aestivum subsp. aestivum	PI57171	CItr 7118	Georgia	White pericarp	TaMYC1w
476	Triticum aestivum subsp. aestivum	PI84527	176	Uzbekistan, Farghona	White pericarp	TaMYC1w
477	Triticum aestivum subsp. aestivum	PI94452	132	Ukraine, Dnipropetrovsk	White pericarp	TaMYC1w
478	Triticum aestivum subsp. aestivum	PI94465	145	Ukraine, Krym	White pericarp	TaMYC1w
479	Triticum aestivum subsp. aestivum	PI105098	Touko	Finland	White pericarp	TaMYC1w
480	Triticum aestivum subsp. aestivum	PI113946	Caesium 111	Russian Federation, Omsk	White pericarp	TaMYC1w
481	Triticum aestivum subsp. aestivum	PI113948	Kooperatorka	Ukraine, Odesa	White pericarp	TaMYC1w
482	Triticum aestivum subsp. aestivum	PI116317	G 0-0-110	Australia, New South Wales	White pericarp	TaMYC1w
483	Triticum aestivum subsp. aestivum	PI118727	124	China, Henan	White pericarp	TaMYC1w
484	Triticum aestivum subsp. aestivum	PI163432		Argentina, Buenos Aires	White pericarp	TaMYC1w
485	Triticum aestivum subsp. aestivum	PI163440		Argentina, Santa Fe	White pericarp	TaMYC1w
486	Triticum aestivum subsp. aestivum	PI164549	8843	India, Madhya Pradesh	White pericarp	TaMYC1w
487	Triticum aestivum subsp. aestivum	PI175517	Sampo	Finland	White pericarp	TaMYC1w
488	Triticum aestivum subsp. aestivum	PI387563	IAR/W/117-3	Ethiopia	White pericarp	TaMYC1w

TABLE S6 | Oligonucleotide primers used in this work.

**Primer**	**Sequence (5'-3')**	**Use**
TaMYC1cdsF	ATGCATATGAAGGAAGGAAA	Cloning *TaMYC1* cDNAs
TaMYC1cdsR	CCAAATTTACGAATCTACGG	
Tubulin-F	TGAGGACTGGTGCTTACCGC	Amplifying wheat tubulin gene transcripts (as internal control of RT-PCR)
Tubulin-R	GCACCATCAAACCTCAGGGA	
TaDFR-F	CTCTCATGGCTCGTCAGGAAG	Amplifying wheat DFR gene transcripts
TaDFR-R	TCTTGGGAGTCGAAGTCCAT	
TaMYC1promoterF	TTTTGCTACTGTTGTGGTCA	Cloning *TaMYC1* promoter region
TaMYC1promoterF	TGGTTGCTGAATTGCGTCCC	
Xtamyc1F	CCAAGAACGAGGGGGAAA	Amplifying the PCR marker *Xtamyc1*
Xtamyc1R	ACGAGTGACTGCGACGCA	
TaMYC1AttB1F	AAAAAGCAGGCTTCATGGCGCTGTCAGTAGTTCG	Construction of pUbi-TaMYC1-I, pUbi-TaMYC1-II, pUbi-TaMYC1-III, pUbi-TaMYC1-IV, pUbi-TaMYC1-V, pUbi-TaMYC1-VI
TaMYC1AttB2R	AAAAAGCAGGCTTCATGGCCTGCGAATAGCTC	
ZmRAttB1F	AAAAAGCAGGCTTCATGGCGCTTTCAGCTTCCCGAG	Construction of pUbi-ZmR
ZmRAttB2R	AGAAAGCTGGGTCTCACCGCTTCCCTATAGCTTTGC	
ZmC1AttB1F	AAAAAGCAGGCTTCATGGGGAGGAGGGCGTGTTG	Construction of pUbi-ZmC1
ZmC1AttB2R	AGAAAGCTGGGTCCTACGCAAGCTGCCCGGCCGT	
attB1 adapter	GGGGACAAGTTTGTACAAAAAAGCAGGCT	Universal *att*B adapter primers
attB2 adapter	GGGGACCACTTTGTACAAGAAAGCTGGGT	
TaMYC1BgliiF	GGAGATCTATGGCGCTGTCAGTAGTTCG	Construction of p35S-TaMYC1-GFP
TaMYC1BgliiR	GGAGATCTGGTGGCCTGCGAATAGCTCTCT	
TaMYC1BSMVF	CGGCTAGCCTGCCGCTGTGAGGAGCAT	Preparing BSMV:TaMYC1as
TaMYC1BSMVR	CGGCTAGCCAGACGGTGTAGTACCATTCG	
BSMVcpF	TGACTGCTAAGGGTGGAGGA	Detection of BSMV *CP* by RT-PCR
BSMVcpR	CGGTTGAACATCACGAAGAGT	
